# A novel ruthenium complex with xanthoxylin induces S-phase arrest and causes ERK1/2-mediated apoptosis in HepG2 cells through a p53-independent pathway

**DOI:** 10.1038/s41419-017-0104-6

**Published:** 2018-01-23

**Authors:** Nanashara C. de Carvalho, Sara P. Neves, Rosane B. Dias, Ludmila de F. Valverde, Caroline B. S. Sales, Clarissa A. G. Rocha, Milena B. P. Soares, Edjane R. dos Santos, Regina M. M. Oliveira, Rose M. Carlos, Paulo C. L. Nogueira, Daniel P. Bezerra

**Affiliations:** 1 Gonçalo Moniz Institute, Oswaldo Cruz Foundation (IGM-FIOCRUZ/BA), Salvador, Bahia 40296-710 Brazil; 2 Department of Biomorphology, Institute of Health Sciences, Federal University of Bahia, Salvador, Bahia 40110-902 Brazil; 3 Center of Biotechnology and Cell therapy, Hospital São Rafael, Salvador, Bahia 41253-190 Brazil; 4 Department of Chemistry, Federal University of São Carlos, São Carlos, São Paulo 13561-901 Brazil; 5 Department of Chemistry, Federal University of Sergipe, São Cristóvão, Sergipe 49100-000 Brazil

## Abstract

Ruthenium-based compounds have gained great interest due to their potent cytotoxicity in cancer cells; however, much of their potential applications remain unexplored. In this paper, we report the synthesis of a novel ruthenium complex with xanthoxylin (RCX) and the investigation of its cellular and molecular action in human hepatocellular carcinoma HepG2 cells. We found that RCX exhibited a potent cytotoxic effect in a panel of cancer cell lines in monolayer cultures and in a 3D model of multicellular cancer spheroids formed from HepG2 cells. This compound is detected at a high concentration in the cell nuclei, induces DNA intercalation and inhibits DNA synthesis, arresting the cell cycle in the S-phase, which is followed by the activation of the caspase-mediated apoptosis pathway in HepG2 cells. Gene expression analysis revealed changes in the expression of genes related to cell cycle control, apoptosis and the MAPK pathway. In addition, RCX induced the phosphorylation of ERK1/2, and pretreatment with U-0126, an MEK inhibitor known to inhibit the activation of ERK1/2, prevented RCX-induced apoptosis. In contrast, pretreatment with a p53 inhibitor (cyclic pifithrin-α) did not prevent RCX-induced apoptosis, indicating the activation of a p53-independent apoptosis pathway. RCX also presented a potent in vivo antitumor effect in C.B-17 SCID mice engrafted with HepG2 cells. Altogether, these results indicate that RCX is a novel anticancer drug candidate.

Hepatocellular carcinoma (HCC) is a primary malignancy of the liver that accounts for most liver cancers, which is also one of the most common cancers in the world. In 2012, HCC was estimated to be responsible for approximately 746,000 deaths worldwide^1^. The antineoplastic chemotherapy for HCC includes doxorubicin, cisplatin and 5-fluorouracil alone or in combination with each other but has low efficacy^2^. More recently, sorafenib, a tyrosine kinase inhibitor, was introduced as the only validated systemic therapy for advanced HCC treatment; however, this treatment prolongs survival by only a mere 3 months. Other tyrosine kinase inhibitors have also been evaluated for HCC but with failed results^3,4^.

Metal complexes have been investigated for cancer treatment since the discovery of the cytotoxic properties of cisplatin, a platinum-based compound^5^. Among them, ruthenium-based compounds have received great interest due to their potent cytotoxic activity in cancer cells^6–9^, and significant progress in the preclinical and clinical development of ruthenium complexes as antineoplastic agents has been observed. These include the development of NAMI-A ([ImH][trans-RuCl_4_(DMSO)(Im)], where Im = imidazole and DMSO = dimethylsulfoxide) and KP1019 ([IndH][trans-RuCl_4_(Ind)_2_], where Ind = indazole), which are in phase I/II clinical trials^10,11^. On the other hand, since the structure of the ligand of the metal-based compounds is related to the cytotoxicity of these complexes, various potentialities of ruthenium complexes remain unexplored.

To obtain additional information about the cytotoxic potential of ruthenium-based compounds, a new ligand, xanthoxylin, was used to synthesize a novel ruthenium complex. Xanthoxylin (2-hydroxy-4,6-dimethoxyacetophenone) is a plant-derived molecule with antibacterial, antifungal, antinociceptive, antiedematogenic and antispasmodic activities^12–15^. In this paper, we report the synthesis of a novel ruthenium complex with xanthoxylin (RCX), *cis*-[Ru(phen)_2_(xant)](PF_6_) (phen = 1,10′-phenanthroline; xant = xanthoxylin) and the investigation of its cellular and molecular action on human hepatocellular carcinoma HepG2 cells.

## Results

### Synthesis of novel ruthenium complex with xanthoxylin

**Fig. 1:**
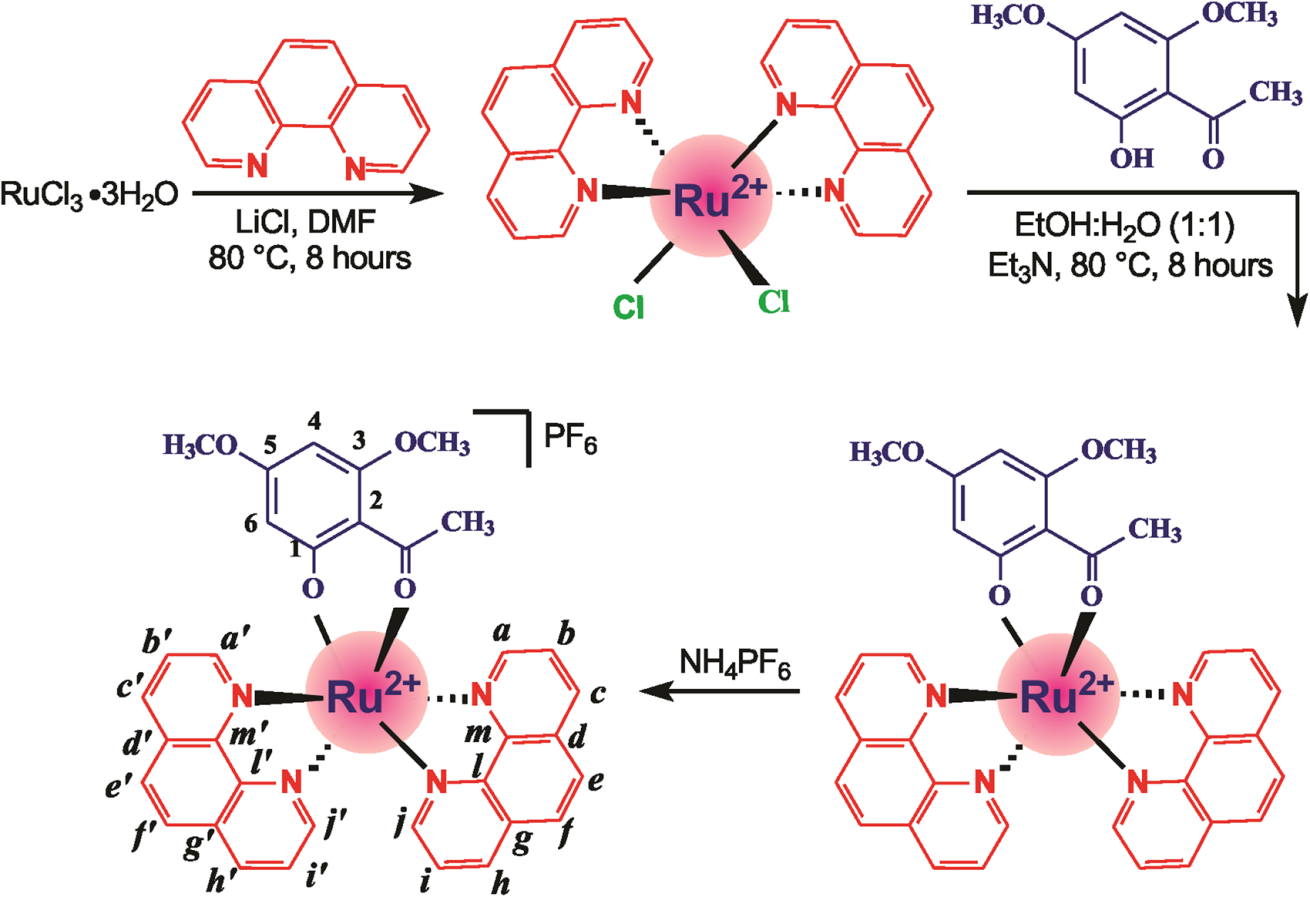
Route for the synthesis of the ruthenium complex with xanthoxylin (RCX), *cis*-[Ru(phen)_2_(xant)](PF_6_) (phen = 1,10´-phenanthroline; xant = xanthoxylin)

**Fig. 2:**
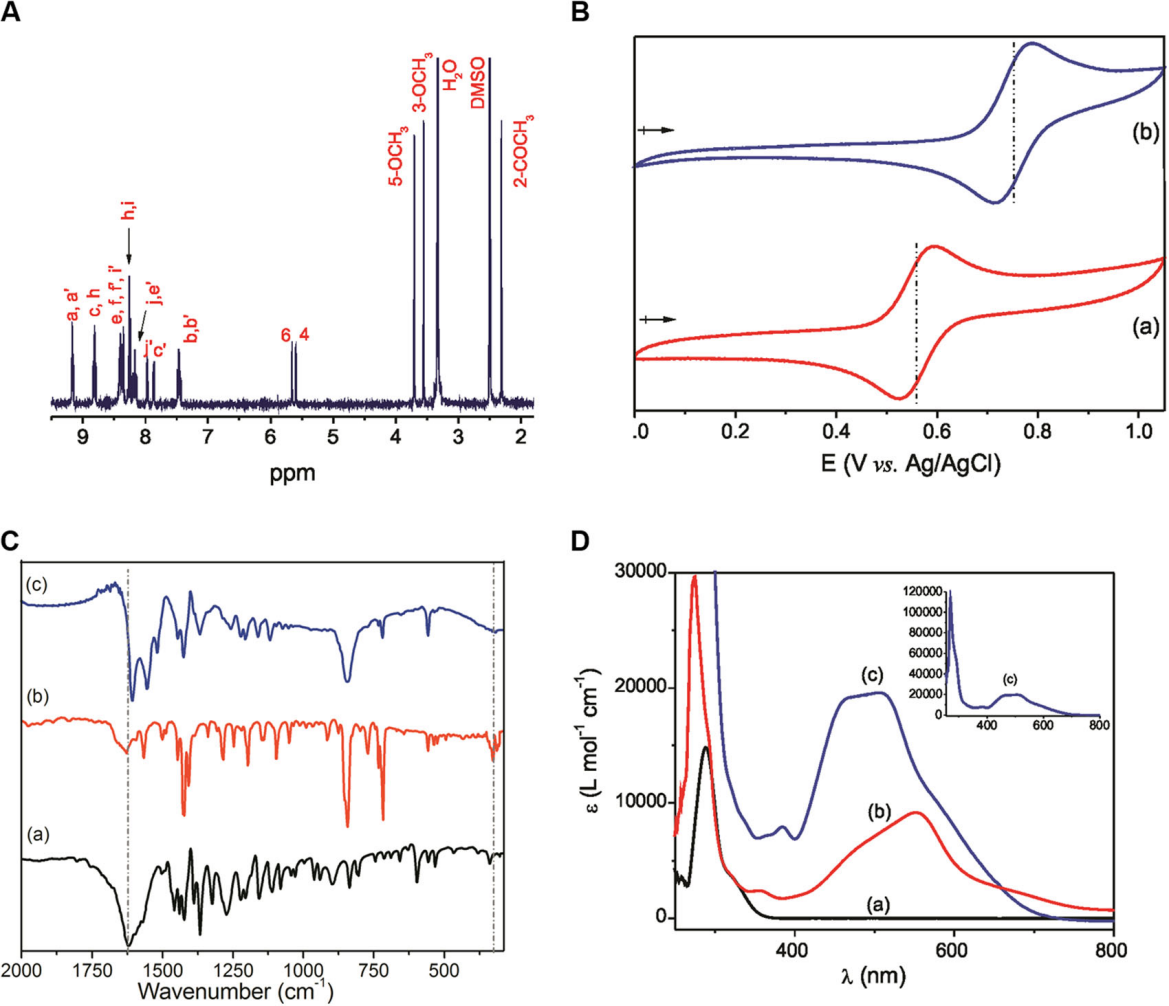
**a** ^1^H NMR spectrum of *cis*-[Ru(phen)_2_(xant)](PF_6_) in DMSO-*d*_6_ at 25 ± 0.1 °C. **b** Cyclic voltammograms in DMF (0.1 M TBAPF_6_) of the following: (a) *cis*-[RuCl_2_(phen)_2_] (precursor) and (b) *cis*-[Ru(phen)_2_(xant)](PF_6_) (RCX). Conditions: complex concentration = 1 mM, Ag/AgCl reference electrode; *v* = 100 mV/s. **c** FTIR spectra of the following at a 1:100 dilution in CsI: (a) xanthoxylin, (b) *cis*-[RuCl_2_(phen)_2_] (precursor) and (c) *cis*-[Ru(phen)(xant)](PF_6_) (RCX). **d** UV-Vis spectra in DMF of the following: (a) xanthoxylin, (b) *cis*-[RuCl_2_(phen)_2_] (precursor) and (c) *cis*-[Ru(phen)(xant)](PF_6_) (RCX)

The RCX was synthesized by reacting a previously deprotonated xanthoxylin with the precursor complex [Ru(phen)_2_Cl_2_] in an ethanol/water mixture using a procedure similar to one already established^16,17^ (Fig. 1). Xanthoxylin is expected to coordinate with the metal center bidentate after deprotonation of the hydroxyl group. The geometry of the formed complex was determined by spectroscopic techniques. The ^1^H nuclear magnetic resonance (NMR) spectrum of RCX was obtained in DMSO-*d*_6_ at 25 °C. The spectrum is shown in Fig. 2a, and the complete assignments are summarized in Table S1. By integrating the area of the hydrogen signals into the ^1^H NMR spectrum of RCX, the signals corresponding to the xanthoxylin and 1,10’-phenanthroline portions were found to be present at a ratio of 1:2, indicating that the Ru(II) ion is hexacoordinated and that chelation occurred through the oxygen atoms of the C1-O^−^ and C1’ = O groups of xanthoxylin and the nitrogen atoms of the two phenanthrolines. In the ^1^H NMR spectrum of RCX, the signal at 13.77 ppm that was attributed to the proton of the 1–OH group of metal-free xanthoxylin is not present. In addition, the signals of the protons 2-COCH_3_, 3-OCH_3_, 5-OCH_3_, 4 and 6 were displaced −0.23, −0.25, −0.15, −0.48 and −0.45 ppm, respectively. Thus, all hydrogen signals of the coordinated xanthoxylin were shifted to a high field. These data are consistent with the redistribution of the xanthoxylin electrons involved in the Ru(II) coordination. These displacements are also consistent with the displacement of the redox potential of RCX, which presents a reversible electron-centered process with E_1/2_ = 751 mV, which is a more positive potential than that observed for the precursor complex *cis*-[RuCl_2_(phen)_2_], E_1/2_ = 562 mV (Fig. 2b). Therefore, the observation that the electron oxidation of RCX is more difficult than that of the precursor is consistent with the increased stabilization of the dπ orbitals of the metal ion, resulting from coordination with xanthoxylin.

The obtained infrared (IR) spectra of free xanthoxylin, precursor and RCX in CsI pellets are shown in Fig. 2c. In the IR spectrum of RCX, the carbonate peak of free xanthoxylin at 1622 cm^−1^ decreased in intensity and was shifted to a lower energy, appearing at 1608 cm^−1^. Additionally, the absence of the peak at 328 cm^−1^ corresponding to the Ru–Cl stretch and the appearance of a new peak at 555 cm^−1^ attributable to the Ru–O stretch is consistent with Ru(II) coordination^18,19^. Additional peaks observed in the 1444–1110 cm^−1^ range are attributed to the CH stretches of the ring deformation of the phenanthrolines and xanthoxylin linkers and at 844 cm^−1^ is attributed to the ν(P–F) mode of the PF_6_^−^ ion, which is consistent with previous studies^20^.

The electronic absorption spectra of free xanthoxylin, the precursor and RCX complex obtained in DMF are shown in Fig. 2d. The intense and broad absorption band observed in the visible region of the precursor complex (*λ*_max_ = 552 nm, *ε* = 8137 mol^−1^ L cm^−1^) and that of RCX (*λ*_max_ = 510 nm, *ε* = 19,555 mol^−1^ L cm^−1^) are qualitatively attributed to the metal-to-ligand charge transfer (MLCT) transition (Ru, dπ → phen, π*). In accordance with the cyclic voltammetric measurements, the energy of RCX MLCT transition was lower than that found for its precursor complex.

### Ruthenium complex with xanthoxylin exhibits potent cytotoxicity in a panel of different cancer cells

The cytotoxicity of RCX in a panel of 15 cancer cell lines and 3 non-cancer cells was evaluated using the Alamar blue assay after a 72 h of incubation. Table 1 shows the IC_50_ (half maximal inhibitory concentration) obtained. RCX presented IC_50_ values of 1.6 and 26.0 μM for the HCT116 and ACP-03 cancer cell lines, respectively, representing the range of IC_50_ values observed. The IC_50_ values for non-cancer cells were 21.3, 6.1 and 4.6 μM for the MRC-5, HaCAT and PBMC cells, respectively. Metal-free xanthoxylin was not cytotoxic to any cells at the concentrations tested (IC_50_ > 127.4 μM). Doxorubicin presented IC_50_ values ranging from 0.2 to 6.8 μM for the HCT116 and ACP-02 cancer cell lines, respectively, and 1.3, 0.1 and 5.2 μM for the non-cancer cells MRC-5, HaCAT and PBMC, respectively. Oxaliplatin presented IC_50_ values ranging from 0.6 to 7.7 μM for the HL-60 and SCC-4 cancer cell lines, respectively, and 9.5 and 9.4 μM for the non-cancer cells MRC-5 and PBMC. Table 2 shows the calculated selectivity index (SI). The SI was calculated using the following formula: SI = IC_50_ [non-cancer cells]/IC_50_ [cancer cells]. Based on the SI, RCX exhibited a selectivity index similar to or higher than that of the positive controls doxorubicin and oxaliplatin, which are clinically useful drugs in the treatment of cancer, in most of the cancer cell lines.

**Table 1:** Cytotoxic activity of the ruthenium complex with xanthoxylin (RCX)

Cells	Histological type	IC_50_ in µM		
		DOX	OXA	RCX
Cancer cells				
HCT116	Human colon carcinoma	0.2	4.1	1.6
		0.1–0.3	2.3–5.5	1.2–2.2
HT-29	Human colon adenocarcinoma	0.3	N.d.	3.4
		0.2–0.4		2.4–4.8
MCF-7	Human breast	1.1	5.9	22.4
	adenocarcinoma	0.3–3.5	3.5–9.9	18.6–27.1
HepG2	Human hepatocellular	1.3	2.2	13.5
	carcinoma	1.0–1.8	1.3–3.8	11.6–15.8
HSC-3	Human oral squamous cell	0.4	3.3	7.7
	carcinoma	0.1–1.2	1.4–7.8	3.2–18.5
SCC-4	Human oral squamous cell carcinoma	2.1	7.7	5.3
		1.7–2.6	4.6–13.0	4.0–7.1
SCC-9	Human oral squamous cell carcinoma	2.6	N.d.	3.4
		2.0–3.3		1.8–6.5
SCC-15	Human oral squamous cell carcinoma	1.5	N.d.	6.8
		0.8–2.9		3.7–11.9
SCC-25	Human oral squamous cell carcinoma	1.0	N.d.	6.5
		0.5–2.3		5.2–8.0
AGP-01	Human ascitic gastric adenocarcinoma	1.9	N.d.	5.3
		0.9–4.3		4.0–7.1
ACP-02	Human gastric adenocarcinoma	6.8	N.d.	18.3
		1.1–24.3		14.2–23.5
ACP-03	Human gastric adenocarcinoma	2.4	N.d.	26.0
		0.9–6.0		18.9–36.0
HL-60	Human promyelocytic leukemia	0.2	0.6	6.6
		0.2–0.3	0.1–0.8	3.7–11.7
K-562	Human chronic myelogenous leukemia	1.0	1.0	6.1
		0.6–1.8	0.1–1.3	3.7–10.0
B16-F10	Murine melanoma	0.6	2.2	12.4
		0.4–0.7	1.2–4.1	9.2–16.8
Non-cancer cells				
MRC-5	Human lung fibroblast	1.3	9.5	21.3
		1.0–1.5	5.7–15.9	17.0–26.7
HaCAT	Human keratinocyte	0.1	N.d.	6.1
		0.02–0.6		3.1–12.0
PBMC	Human peripheral blood mononuclear cells	5.2	9.4	4.6
		2.4–11.4	6.5–11.4	2.3–9.1

Data are presented as IC_50_ values in μM with respective 95% confidence interval obtained by nonlinear regression from atleast three independent experiments performed in duplicate, measured by alamar blue assay after 72 h of incubation. Doxorubicin (DOX) and oxaliplatin (OXA) were used as positive controls. *N.d.* not determined

**Table 2:** Selectivity index of the ruthenium complex with xanthoxylin (RCX)

Cancer cells	Non-cancer cells								
	MRC5			HaCAT			PBMC		
	DOX	OXA	RCX	DOX	OXA	RCX	DOX	OXA	RCX
HCT116	6.5	2.3	13.3	0.5	N.d.	3.8	26	2.3	2.9
HT-29	4.3	N.d.	6.3	0.3	N.d.	1,8	17.3	N.d.	1.4
MCF-7	1.2	1.6	1	0.1	N.d.	0.3	4.7	1.6	0.2
HepG2	1	4.3	1.6	0.1	N.d.	0.5	4	4.3	0.3
HSC-3	3.3	2.9	2.8	0.3	N.d.	0.8	13	2.8	0.6
SCC-4	0.6	1.2	4	0.1	N.d.	1.2	2.5	1.2	0.9
SCC-9	0.5	N.d.	6.3	0.04	N.d.	1.8	2	N.d.	1.4
SCC-15	0.9	N.d.	3.1	0.1	N.d.	0.9	3.5	N.d.	0.7
SCC-25	1.3	N.d.	3.3	0.1	N.d.	0.9	5.2	N.d.	0.7
AGP-01	0.7	N.d.	4	0.1	N.d.	1.2	2.7	N.d.	0.9
ACP-02	0.2	N.d.	1.2	0.02	N.d.	0.3	0.8	N.d.	0.3
ACP-03	0.5	N.d.	0.8	0.04	N.d.	0.2	2.2	N.d.	0.2
HL-60	6.5	15.8	3.2	0.5	N.d.	0.9	26	15.7	0.7
K-562	1.3	9.5	3.5	0.1	N.d.	1	5.2	9.4	0.8
B16-F10	2.2	4.3	1.7	0.2	N.d.	0.5	8.7	4.3	0.4

Data are presented as the selectivity index (SI) calculated using the following formula: SI = IC_50_[non-cancer cells]/IC_50_[cancer cells]. Cancer cells: HCT116 (human colon carcinoma); HT-29 (human colon adenocarcinoma); MCF7 (human breast adenocarcinoma); HepG2 (human hepatocellular carcinoma); HSC-3 (human oral squamous cell carcinoma); SCC-4 (human oral squamous cell carcinoma); SCC-9 (human oral squamous cell carcinoma); SCC-15 (human oral squamous cell carcinoma); SCC-25 (human oral squamous cell carcinoma); AGP-01 (human ascitic gastric adenocarcinoma), ACP-02 (human gastric adenocarcinoma), ACP-03 (human gastric adenocarcinoma), HL-60 (human promyelocytic leukemia); K-562 (human chronic myelogenous leukemia); and B16-F10 (murine melanoma). Non-cancer cells: MRC-5 (human lung fibroblast), HaCAT (human keratinocyte) and PBMC (human peripheral blood mononuclear cells). Doxorubicin (DOX) and oxaliplatin (OXA) were used as positive controls. *N.d.* not determined

**Fig. 3:**
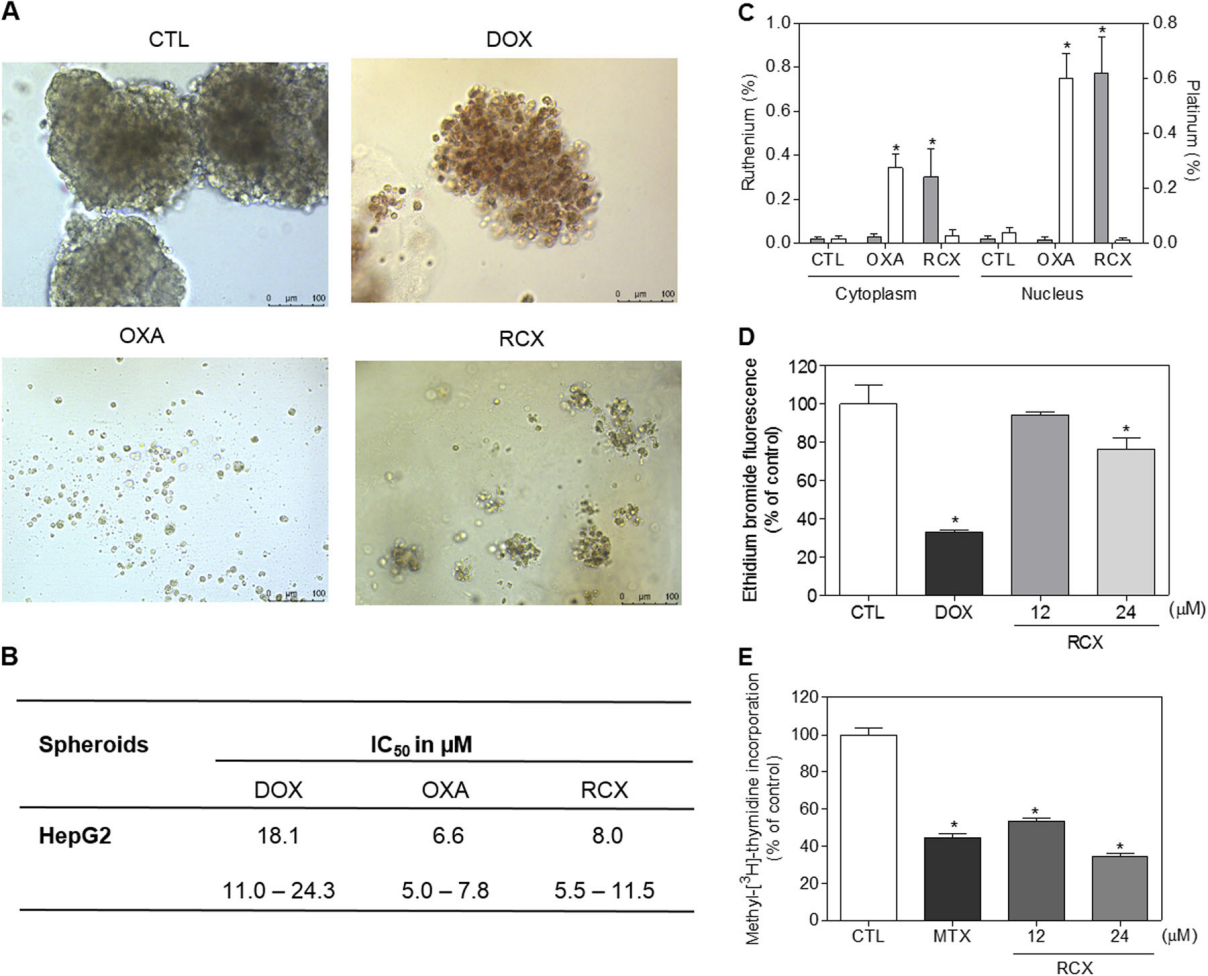
Effect of the ruthenium complex with xanthoxylin (RCX) on a 3D in vitro model of cancer multicellular spheroids formed from HepG2 cells, ruthenium subcellular distribution, and the RCX-induced DNA intercalation and inhibition of DNA synthesis **a** Cells in the 3D in vitro model were examined by light microscopy (bar = 100 µm). **b** IC_50_ values in μM 72 h after incubation with the 3D in vitro model and their respective 95% confidence interval obtained by nonlinear regression from three independent experiments performed in duplicate, as measured by alamar blue assay. The negative control (CTL) was treated with the vehicle (0.2% DMSO) used for diluting the tested compound. Doxorubicin (DOX) and oxaliplatin (OXA) were used as positive controls. **c** Ruthenium subcellular distribution was determined with an energy dispersive X-ray spectrometer in HepG2 cells after 3 h of treatment with 250 µM RCX. Cells without treatment were used as the negative control (CTL). Oxaliplatin (OXA, 500 µM) was used as the positive control, and platinum subcellular distribution was determined. The gray bars represent the percent of ruthenium, and the white bars represent the percent of platinum. Ten cells were analyzed in each treatment. **d** DNA intercalation with RCX was examined by the ability of RCX to displace ethidium bromide from calf thymus DNA. The negative control (CTL) was treated with the vehicle (0.2% DMSO) used for diluting the tested compound. Doxorubicin (DOX, 20 µM) was used as the positive control. **e** DNA synthesis quantification was determined by methyl-[^3^H]-thymidine in HepG2 cells after a 3 h of incubation. The negative control (CTL) was treated with the vehicle (0.2% DMSO) used for diluting the tested compound. Mitoxantrone (MTX, 12 µM) was used as the positive control. Data are presented as the means ± S.E.M. of three independent experiments performed in duplicate. * *P < *0.05 compared with the negative control by ANOVA, followed by the Student–Newman–Keuls test

The cytotoxic effect of RCX was also evaluated with an in vitro three-dimensional (3D) model of cancer using multicellular spheroids formed from HepG2 cells. The morphological changes of the spheroids treated with RCX indicated drug permeability into the 3D culture (Fig. 3a). The IC_50_ value of RCX was 8.0 μM after a 72 h of incubation (Fig. 3b). Doxorubicin and oxaliplatin had IC_50_ values of 18.1 and 6.6 μM, respectively. Therefore, the human hepatocellular carcinoma HepG2 cell line was used as a cellular model for further experiments.

After incubating with RCX for 12, 24, 48 or 72 h, HepG2 cell viability was determined by the trypan blue exclusion (TBE) assay (Fig. S1). At the concentrations of 12 and 24 μM, RCX reduced the number of viable cells by 57.2% and 62.2%, respectively, after 24 h, 74.6% and 73.9% after 48 h, and 73.8% and 82.1% after 72 h. No significant decrease in the number of viable cells was observed after the 12 h of incubation. In addition, RCX induced a significant increase in the number of non-viable cells only after the 48 and 72 h of incubations. Doxorubicin and oxaliplatin also reduced the number of viable cells after incubation for 24 h. Doxorubicin also induced a significant increase in the number of non-viable cells after a 72 h of incubation.

### Ruthenium complex with xanthoxylin induces DNA intercalation and inhibits DNA synthesis

The subcellular distribution of ruthenium was assessed with an energy dispersive X-ray spectrometer (EDS) in RCX-treated HepG2 cells after a 3 h of incubation. Oxaliplatin, a positive control, was used to determine the subcellular platinum distribution. Similar to oxaliplatin, RCX was detected in both the cytoplasm and nucleus of HepG2 cells but was at the highest concentration in the cell nucleus (Fig. 3c). Thus, we decided to investigate if RCX is able to induce DNA intercalation. DNA intercalation was evaluated in a cell-free system using calf thymus DNA (ctDNA) as a model. For this, RCX was added to a DNA-ethidium bromide mixture to examine its ability to displace ethidium bromide from the DNA, thereby decreasing the fluorescence intensity of ethidium bromide. At 20 μM, RCX treatment decreased the ethidium bromide fluorescence (*P* < 0.05), indicating that RCX intercalates with DNA (Fig. 3d). Doxorubicin, a known DNA intercalator, was used at 20 μM as the positive control and also significantly reduced the fluorescence intensity.

Since RCX was able to induce DNA intercalation, we investigated if it interferes with DNA synthesis in HepG2 cells. DNA synthesis was measured by the incorporation of methyl-[^3^H]-thymidine after a 3 h of incubation. At the concentrations of 12 and 24 μM, RCX reduced DNA synthesis in HepG2 cells by 46.4% and 65.2% (*P* < 0.05) (Fig. 3e), respectively. Mitoxantrone, a known DNA synthesis inhibitor, was used as the positive control and reduced DNA synthesis by 55.2% at 12 μM.

### Ruthenium complex with xanthoxylin causes S-phase arrest in HepG2 cells

The effect of RCX on cell cycle progression and internucleosomal DNA fragmentation of HepG2 cells after 12, 24, 48 or 72 h of incubation were evaluated using flow cytometry. Table 3 shows the cell cycle distribution. All DNA that was subdiploid (sub-G_0_/G_1_) was considered fragmented. At a concentration of 12 µM and after a 24 h of incubation, RCX treatment resulted in a significant increase in the number of cells in S-phase compared to the number of cells in S-phase in the negative control (*P < *0.05). In addition to the increase in the number of cells in S-phase, an increase in the amount of internucleosomal DNA fragmentation was also observed for both tested concentrations after a 12 h of incubation (*P < *0.05). Doxorubicin and oxaliplatin caused cell cycle arrest at the G_2_/M phase, which was followed by internucleosomal DNA fragmentation.

**Table 3:** Effect of the ruthenium complex with xanthoxylin (RCX) in the cell cycle distribution of HepG2 cells

Treatment	Concentration (µM)	DNA content (%)			
		Sub-G_0_/G_1_	G_0_/G_1_	S	G_2_/M
12 h of incubation					
CTL	–	4.4 ± 0.7	55.2 ± 2.0	13.3 ± 1.2	17.6 ± 1.3
DOX	2	13.4 ± 1.2*	51.9 ± 4.8	6.9 ± 0.1	22.6 ± 2.6
OXA	10	6.2 ± 0.7	44.4 ± 4.7	12.9 ± 4.4	43.5 ± 3.4*
RCX	12	9.6 ± 1.1*	66.4 ± 4.0	18.0 ± 7.4	13.3 ± 1.1
	24	13.0 ± 2.5*	68.9 ± 2.2	8.6 ± 4.5	13.9 ± 1.6
24 h of incubation					
CTL	–	1.2 ± 0.2	58.9 ± 0.9	15.7 ± 0.3	20.5 ± 1.2
DOX	2	16.8 ± 4.3*	20.9 ± 3.3*	8.9 ± 2.3*	66.9 ± 5.5*
OXA	10	4.8 ± 0.5	26.0 ± 1.1*	18.5 ± 1.4	47.1 ± 0.7*
RCX	12	15.3 ± 0.6*	38.2 ± 1.2*	22.4 ± 0.7*	17.6 ± 0.9
	24	34.7 ± 1.4*	29.9 ± 2.7*	13.0 ± 1.6	13.0 ± 1.3
48 h of incubation					
CTL	–	1.2 ± 0.5	61.9 ± 1.2	14.8 ± 1.2	14.9 ± 0.5
DOX	2	20.9 ± 1.0*	34.6 ± 2.7*	9.1 ± 1.9	31.1 ± 3.3*
OXA	10	6.8 ± 1.3	34.7 ± 3.7*	21.2 ± 1.7*	33.7 ± 1.5*
RCX	12	44.1 ± 6.3*	23.3 ± 2.0*	11.5 ± 2.7	9.0 ± 0.8*
	24	62.6 ± 3.4*	18.9 ± 3.0*	10.3 ± 1.3	6.5 ± 0.7*
72 h of incubation					
CTL	–	3.6 ± 0.7	64.4 ± 2.1	12.4 ± 1.1	13.1 ± 1.4
DOX	2	46.6 ± 2.8*	31.6 ± 5.1*	12.5 ± 1.1	16.3 ± 1.1
OXA	10	17.6 ± 3.9*	30.1 ± 5.1*	16.2 ± 1.1	38.0 ± 5.4*
RCX	12	42.6 ± 6.8*	23.6 ± 1.9*	13.2 ± 0.8	11.1 ± 1.8
	24	68.2 ± 3.7*	19.7 ± 3.3*	4.9 ± 1.1*	6.7 ± 0.7

Data are presented as the means ± S.E.M. of three independent experiments performed in duplicate. The negative control (CTL) was treated with the vehicle (0.2% DMSO) used for diluting the tested compound. Doxorubicin (DOX) and oxaliplatin (OXA) were used as positive controls. Ten thousand events were evaluated per experiment and cellular debris was omitted from the analysis. * *P *<  0.05 compared with the negative control by ANOVA, followed by the Student–Newman–Keuls Test

### Ruthenium complex with xanthoxylin triggers caspase-mediated apoptosis of HepG2 cells

**Fig. 4:**
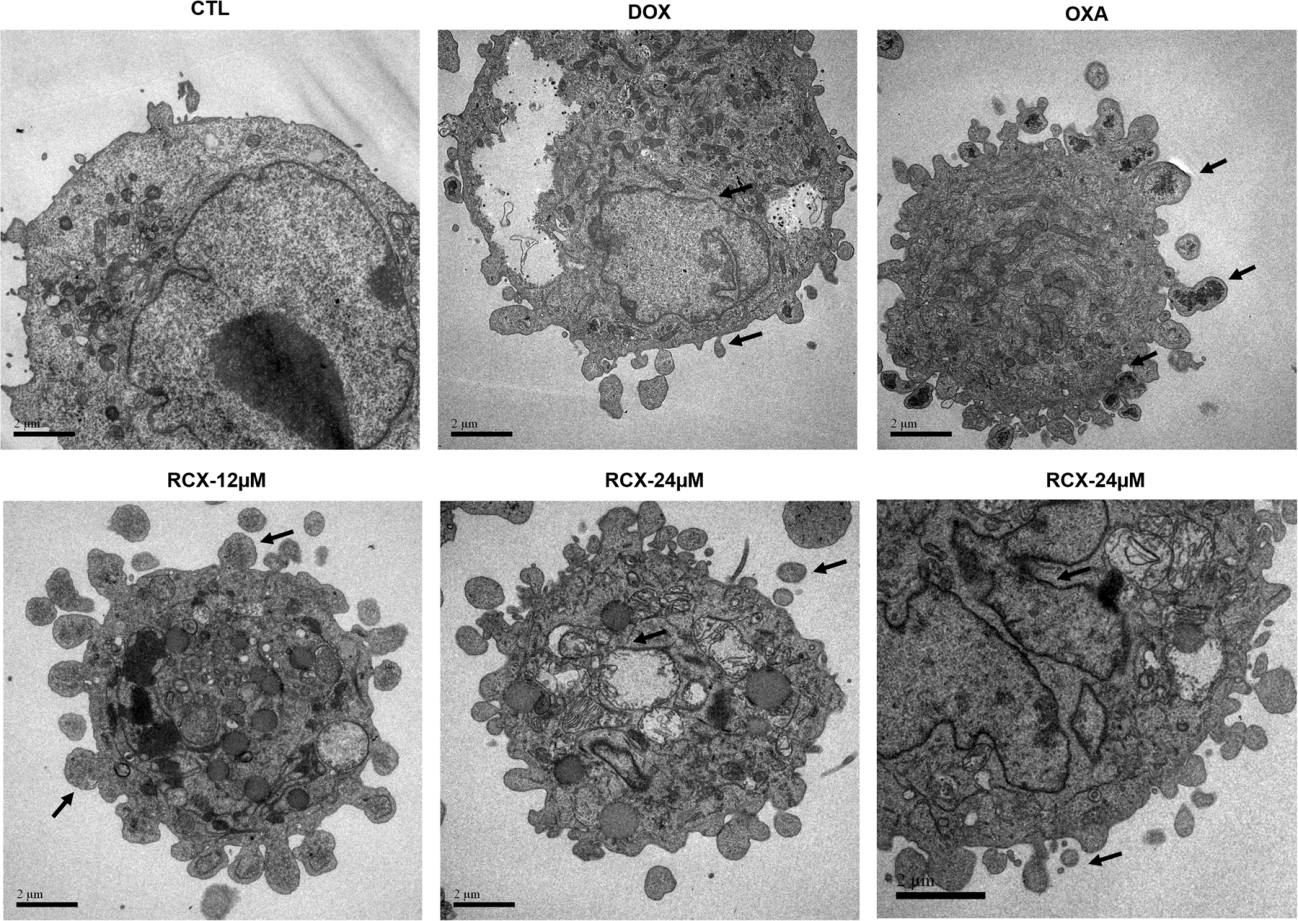
Effect of the ruthenium complex with xanthoxylin (RCX) on the morphological analysis of HepG2 cells after 24 h of incubation, as examined by a transmission electron microscope Arrows indicate cells with nuclear fragmentation, the presence of membrane bubbles and/or apoptotic bodies (bar = 2 µm). The negative control (CTL) was treated with the vehicle (0.2% DMSO) used for diluting the tested compound. Doxorubicin (DOX, 2 µM) and oxaliplatin (OXA, 10 µM) were used as positive controls

Using light microscopy to analyze cell morphology, we observed cell shrinkage, chromatin condensation and nuclear fragmentation in cells treated with RCX (Fig. S2). Additionally, flow cytometric analysis also showed cell shrinkage caused by RCX, as observed by a decrease in forward light scatter (FSC), and nuclear condensation, as observed by a transient increase in side scatter (SCC), indicating morphological changes consistent with apoptosis (Fig. S3). Furthermore, transmission electron microscope (TEM) analysis indicated cell shrinkage, nuclear fragmentation, the presence of membrane bubbles and apoptotic bodies (Fig. 4). Doxorubicin and oxaliplatin treatment also led to an apoptotic morphology.

**Fig. 5:**
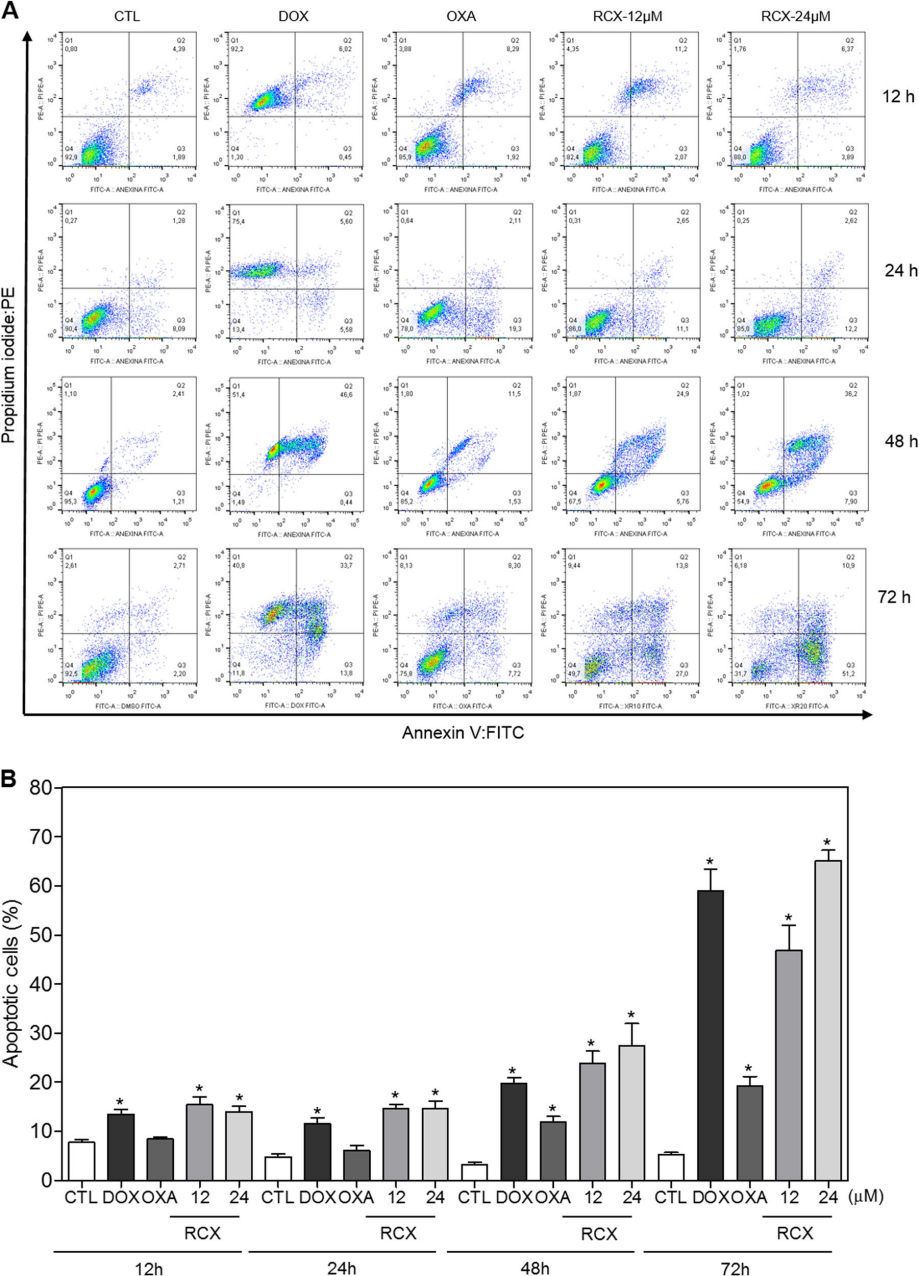
Effect of the ruthenium complex with xanthoxylin (RCX) on the induction of apoptosis in HepG2 cells after 12, 24, 48 or 72 h of incubation, as determined by flow cytometry using annexin V-FITC/PI staining **a** Representative flow cytometry dot plots show the percent cells in the viable, early apoptotic, late apoptotic and necrotic stages. **b** Quantification of apoptotic HepG2 cells. The negative control (CTL) was treated with the vehicle (0.2% DMSO) used for diluting the tested compound. Doxorubicin (DOX, 2 µM) and oxaliplatin (OXA, 10 µM) were used as positive controls. Data are presented as the means ± S.E.M. of three independent experiments performed in duplicate. Ten thousand events were evaluated per experiment, and cellular debris was omitted from the analysis. * *P* < 0.05 compared with the negative control by ANOVA, followed by the Student–Newman–Keuls test

**Fig. 6:**
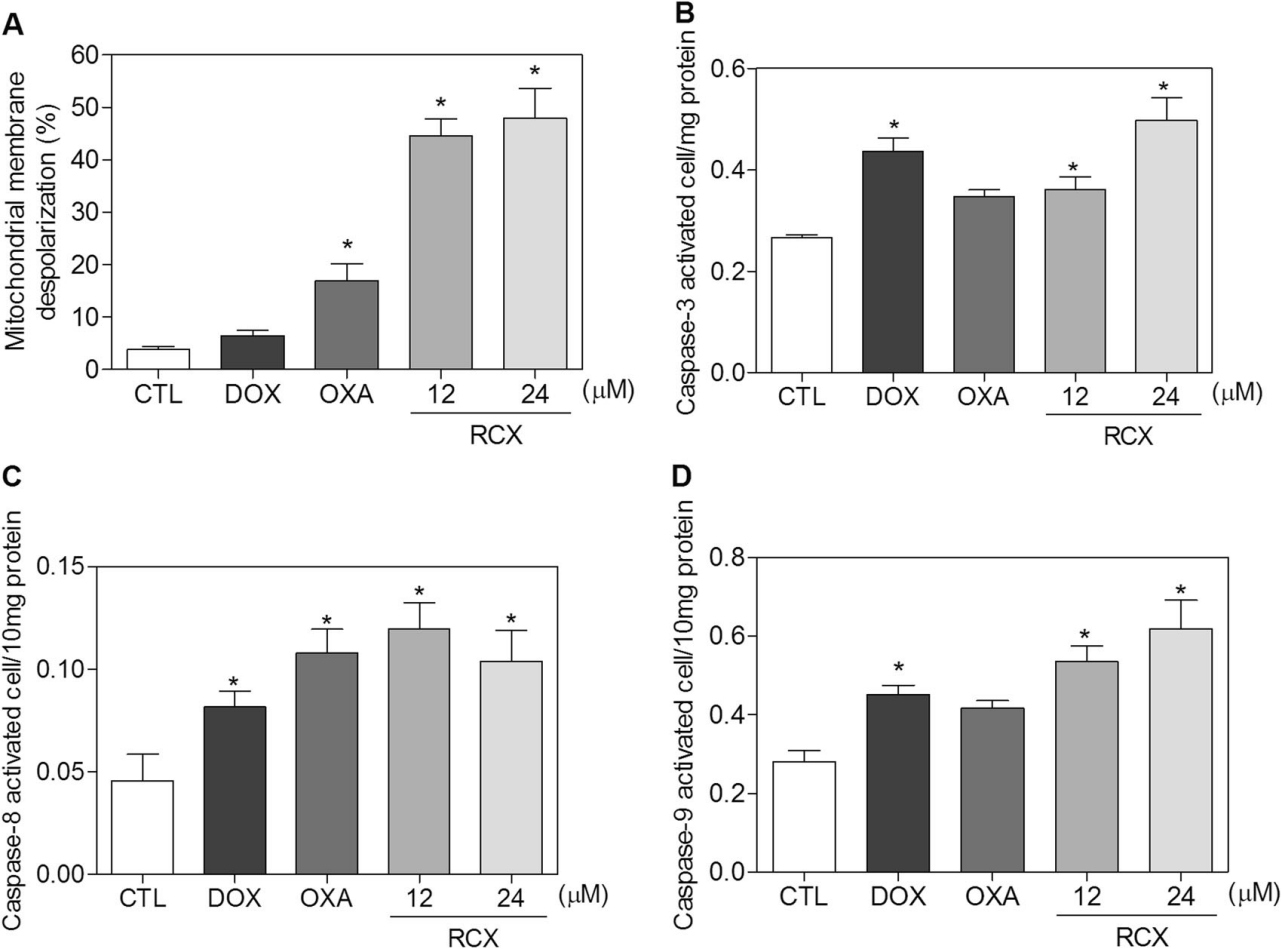
Effect of the ruthenium complex with xanthoxylin (RCX) on the mitochondrial membrane potential and caspase-3, -8 and -9 activity in HepG2 cells **a** Mitochondrial membrane potential was determined by flow cytometry using rhodamine 123 staining after 24 h of incubation with RCX. **b** Caspase-3 activity was determined by colorimetric assay after 48 h of incubation with RCX. **c** Caspase-8 activity was determined by colorimetric assay after 48 h of incubation with RCX. **d** Caspase-9 activity was determined by colorimetric assay after 48 h of incubation with RCX. The negative control (CTL) was treated with the vehicle (0.2% DMSO) used for diluting the tested compound. Doxorubicin (DOX, 2 µM) and oxaliplatin (OXA, 10 µM) were used as positive controls. Data are presented as the means ± S.E.M. of three independent experiments performed in duplicate. For flow cytometry analysis, 10,000 events were evaluated per experiment, and cellular debris was omitted from the analysis. * *P* < 0.05 compared with the negative control by ANOVA, followed by the Student–Newman–Keuls test

**Fig. 7:**
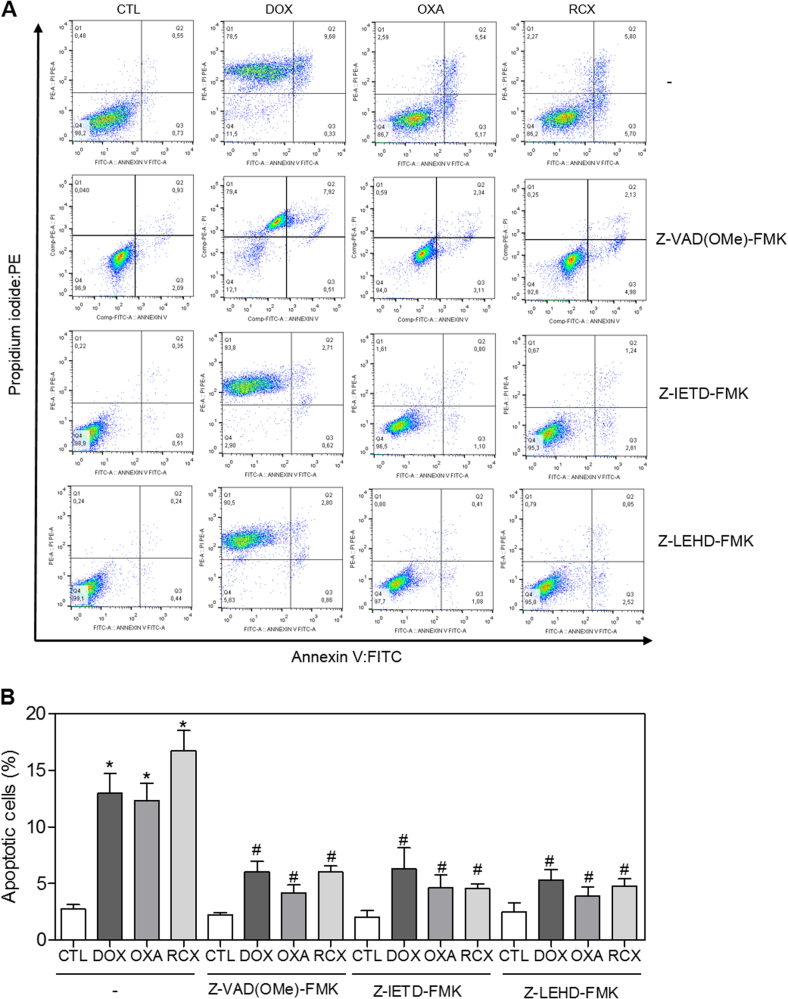
Effects of the pan-caspase (Z-VAD(Ome)-FMK), caspase-8 (Z-IETD-FMK) and caspase-9 (Z-LEHD-FMK) inhibitors in the apoptosis induced by the ruthenium complex with xanthoxylin (RCX) in HepG2 cells, determined by flow cytometry using annexin V-FITC/PI staining **a** Representative flow cytometry dot plots showing the percent of cells in the viable, early apoptotic, late apoptotic and necrotic stages. **b** Quantification of apoptotic HepG2 cells. For protection assays, the cells were pretreated for 2 h with 50 µM Z-VAD(Ome)-FMK, 20 µM Z-IETD-FMK or 20 µM Z-LEHD-FMK and then incubated with 12 µM RCX for 48 h. The negative control (CTL) was treated with the vehicle (0.2% DMSO) used for diluting the tested compound. Doxorubicin (DOX, 2 µM) and oxaliplatin (OXA, 10 µM) were used as positive controls. Data are presented as the means ± S.E.M. of three independent experiments performed in duplicate. Ten thousand events were evaluated per experiment, and cellular debris was omitted from the analysis. * *P* < 0.05 compared with the negative control by ANOVA, followed by the Student–Newman–Keuls test. # *P* < 0.05 compared with the respective treatment without inhibitor by ANOVA, followed by the Student–Newman–Keuls test

In addition to the morphological analysis, we performed Annexin V/propidium iodide (PI) double staining in HepG2 cells after 12, 24, 48 or 72 h of incubation, and the numbers of viable, early apoptotic, late apoptotic and necrotic cells were quantified (Fig. 5). RCX caused apoptosis in a time- and concentration-dependent manner. No significant increase in the number of necrotic cells was observed. Using flow cytometry after a 24 h of incubation, RCX also induced mitochondrial depolarization in HepG2 cells, as measured by the incorporation of rhodamine 123 (Fig. 6a). Next, we studied the activation of the effector (caspase-3) and initiator (caspases-8 and -9) caspases on RCX-treated HepG2 cells after 48 h of incubation. Incubation with RCX caused the activation of all caspases analyzed (Figs. 6b, c, d), indicating the activation of the caspase-mediated apoptosis pathway. Moreover, pretreatment with a pan-caspase inhibitor (Z-VAD(OMe)-FMK), caspase-8 inhibitor (Z-IETD-FMK) or caspase-9 inhibitor (Z-LEHD-FMK) prevented the RCX-induced apoptosis (Figs. 7a, b).

The cytotoxic activity of RCX in wild-type immortalized mouse embryonic fibroblasts (WT SV40 MEF) and immortalized mouse embryonic fibroblast with the BAD gene knocked out (BAD KO SV40 MEF) was also evaluated by the alamar blue assay after a 72 h of incubation. The IC_50_ values for RCX were 3.9 μM for the WT SV40 MEF cell line and 4.1 μM for the BAD KO SV40 MEF cell line, suggesting that BAD is not essential for RCX-induced cytotoxicity. Doxorubicin presented with IC_50_ values of 0.04 and 0.41 μM, while 5-fluorouracil presented with IC_50_ values of 1.7 and 7.3 μM in WT SV40 MEF and BAD KO SV40 MEF cell lines, respectively.

The effect of RCX on intracellular reactive oxygen species (ROS) levels was also investigated in HepG2 cells through flow cytometry using the redox-sensitive fluorescent probe 2′,7′-dichlorofluorescin diacetate (DCF-DA). However, RCX did not induce a significant increase in ROS levels after 1 or 3 h of incubation (Fig. S4A). In addition, pretreatment with the antioxidant N-acetyl-L-cysteine (NAC) did not prevent the reduction in the number of viable cells, as assessed by TBE assay after 24 h of incubation (Fig. S4B).

### Ruthenium complex with xanthoxylin alters gene expression in HepG2 cells

Out of a total of 94 genes investigated, 40 up-regulated and 2 down-regulated genes were identified in HepG2 cells treated with 12 µM RCX for a 12 h of incubation (Table 4 and S2). Among them, the pro-apoptotic genes BCL2L11 (RQ = 9.8 for RCX and RQ = 14.0 for doxorubicin), FAS (RQ = 2.9 for RCX and RQ = 2.5 for doxorubicin) and E2F1 (RQ = 7.1 for RCX and RQ = 4.8 for doxorubicin) were up-regulated. A gene related to cell cycle control (G_1_/S transition), CDKN2A (RQ = 3.8 for RCX and RQ = 2.0 for doxorubicin), was up-regulated. Moreover, at least seven genes related to the mitogen-activated protein kinase (MAPK) pathway, including BRAF (RQ = 2.3 for RCX and RQ = 1.0 for doxorubicin), ELK1 (RQ = 5.2 for RCX and RQ = 2.1 for doxorubicin), JUN (RQ = 4.5 for RCX and RQ = 7.3 for doxorubicin), MAP2K1 (RQ = 2.6 for RCX and RQ = 2.4 for doxorubicin), MAPK1 (RQ = 2.1 for RCX and RQ = 0.9 for doxorubicin), MAPK14 (RQ = 3.0 for RCX and RQ = 2.2 for doxorubicin) and RAF1 (RQ = 4.7 for RCX and RQ = 3.5 for doxorubicin), were up-regulated, indicating a substantial role for MAPK signaling in the action of RCX in HepG2 cells. In contrast, the main gene involved in p53 pathway activation, TP53 (RQ = 0.5 for RCX and RQ = 1.0 for doxorubicin), was down-regulated, whereas the gene related to the inactivation of the p53 pathway MDM2 (RQ = 3.2 for RCX and RQ = 0.9 for doxorubicin) was up-regulated.

**Table 4:** Effect of the ruthenium complex with xanthoxylin (RCX) in gene expression of HepG2 cells

Symbol	Full name	RQ	
		DOX	RCX
**Genes up-regulated**			
AKT2	AKT serine/threonine kinase 2	3.3	2.7
BCAR1	BCAR1, Cas family scaffolding protein	2.2	2.9
BCL2	BCL2, apoptosis regulator	1.1	2.4
BCL2L11	BCL2 like 11	14.0	9.8
BRAF	B-Raf proto-oncogene, serine/threonine kinase	1.0	2.3
CCND1	Cyclin D1	0.8	4.8
CCND3	Cyclin D3	1.2	2.4
CDC42	Cell division cycle 42	1.1	4.2
CDK4	Cyclin dependent kinase 4	1.4	2.7
CDKN2A	Cyclin dependent kinase inhibitor 2A	2.0	3.8
CRK	CRK proto-oncogene, adaptor protein	2.5	6.2
DVL1	Dishevelled segment polarity protein 1	0.5	2.7
E2F1	E2F transcription factor 1	4.8	7.1
EGFR	Epidermal growth factor receptor	0.5	2.0
ELK1	ELK1, ETS transcription factor	2.1	5.2
ERBB2	erb-b2 receptor tyrosine kinase 2	1.8	2.2
FAS	Fas cell surface death receptor	2.5	2.9
FGF2	Fibroblast growth factor 2	1.9	5.5
FYN	FYN proto-oncogene, Src family tyrosine kinase	1.0	2.2
FZD1	Frizzled class receptor 1	5.8	4.8
HRAS	HRas proto-oncogene, GTPase	1.4	2.1
IGF1	Insulin like growth factor 1	N.d.	N.d.
ITGA2B	Integrin subunit alpha 2b	2.4	5.4
JUN	Jun proto-oncogene, AP-1 transcription factor subunit	7.3	4.5
KRAS	KRAS proto-oncogene, GTPase	2.6	4.8
MAP2K1	Mitogen-activated protein kinase kinase 1	2.4	2.6
MAPK1	Mitogen-activated protein kinase 1	0.9	2.1
MAPK14	Mitogen-activated protein kinase 14	2.2	3.0
MDM2	MDM2 proto-oncogene	0.9	3.2
MYC	MYC proto-oncogene, bHLH transcription factor	0.4	4.3
NFKB1	Nuclear factor kappa B subunit 1	2.0	2.2
NFKB2	Nuclear factor kappa B subunit 2	3.6	3.6
PTK2	Protein tyrosine kinase 2	1.0	2.0
RAF1	Raf-1 proto-oncogene, serine/threonine kinase	3.5	4.7
RELA	RELA proto-oncogene, NF-kB subunit	1.1	2.4
SHC1	SHC adaptor protein 1	1.9	3.7
SMAD4	SMAD family member 4	2.6	3.6
SOS1	SOS Ras/Rac guanine nucleotide exchange factor 1	2.2	5.0
TCF3	Transcription factor 3	1.9	3.2
VEGFA	Vascular endothelial growth factor A	1.8	3.8
**Genes down-regulated**			
CDKN1B	Cyclin dependent kinase inhibitor 1B	1.0	0.5
TP53	Tumor protein p53	1.0	0.5

HepG2 cells were treated with 13 µM of RCX for 12 h. The negative control was treated with the vehicle (0.2% DMSO) used for diluting the tested compound. Doxorubicin (DOX, 2 µM) was used as positive control. After treatment, total RNA was isolated and reverse transcribed. Gene expression was detected using the 96-well plate TaqMan® Array Human Molecular Mechanisms of Cancer. GAPDH, 18S and HPRT1 genes were used as endogenous genes for normalization. Values represent the relative quantitation (RQ) compared with the calibrator (cells treated with the negative control, RQ = 1.0). The genes were considered to be up-regulated if RQ ≥ 2 and were considered to be down-regulated if RQ ≤ 0.5. *N.d*. not determined.

### Ruthenium complex with xanthoxylin causes ERK1/2-mediated apoptosis in HepG2 cells through a p53-independent pathway

**Fig. 8:**
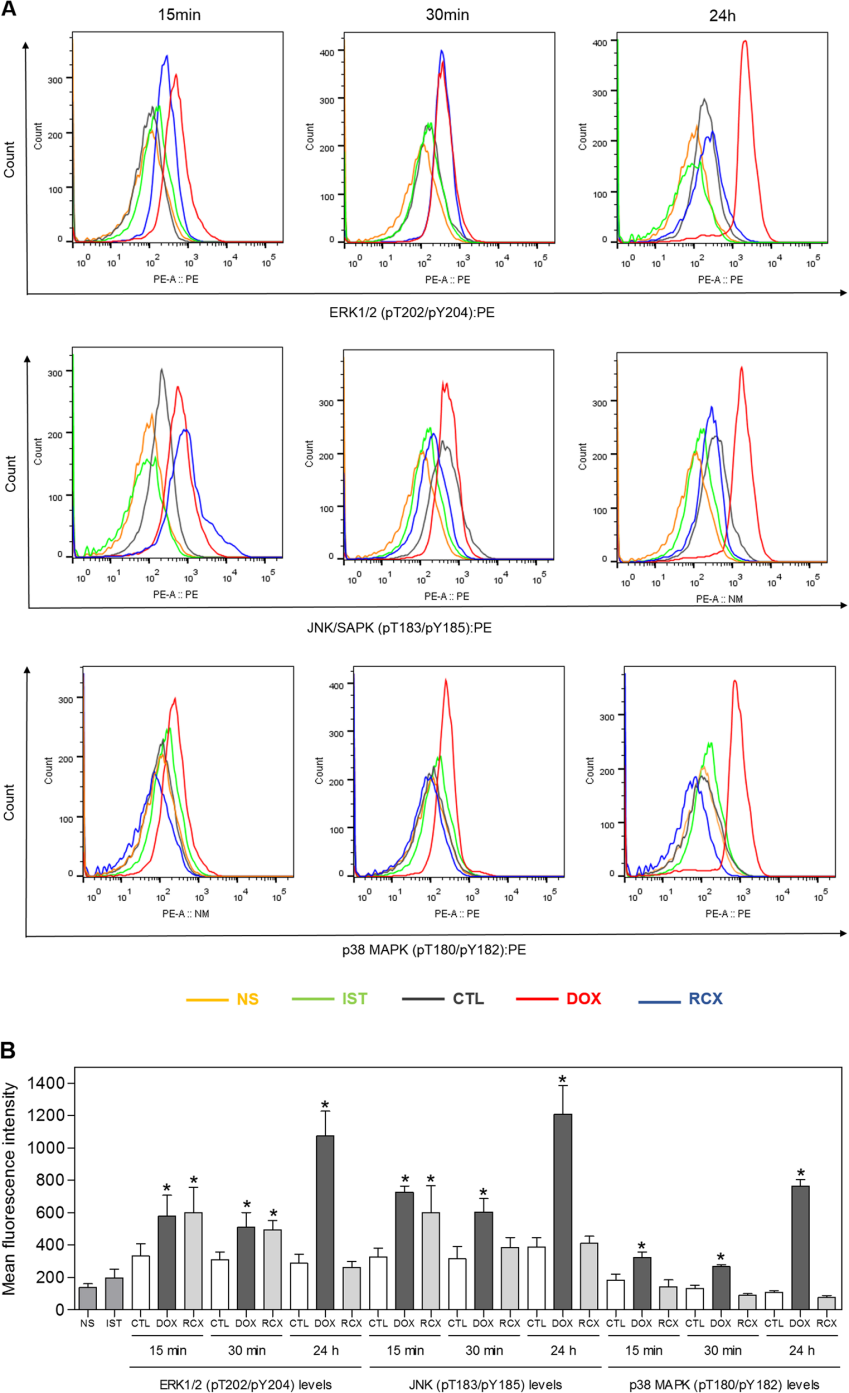
Effect of ruthenium complex with xanthoxylin (RCX) on ERK1/2 (pT202/pY204), JNK/SAPK (pT183/pY185) and p38 MAPK (pT180/pY182) levels, as determined by Phosflow analysis of HepG2 cells treated with 12 µM RCX for an acute (15 or 30 min) or prolonged (24 h) incubation **a** Representative flow cytometry histograms. **b** Quantification of ERK1/2 (pT202/pY204), JNK/SAPK (pT183/pY185) and p38 MAPK (pT180/pY182) levels. The negative control (CTL) was treated with the vehicle (0.2% DMSO) used for diluting the tested compound. Doxorubicin (DOX, 2 µM) was used as the positive control. Data are presented as the means ± S.E.M. of three independent experiments performed in duplicate. Ten thousand events were evaluated per experiment, and cellular debris was omitted from the analysis. * *P* < 0.05 compared with the negative control by ANOVA, followed by the Student–Newman–Keuls test. *NS* Non-stained cells (basal cell fluorescence); *IST* Isotype control

**Fig. 9:**
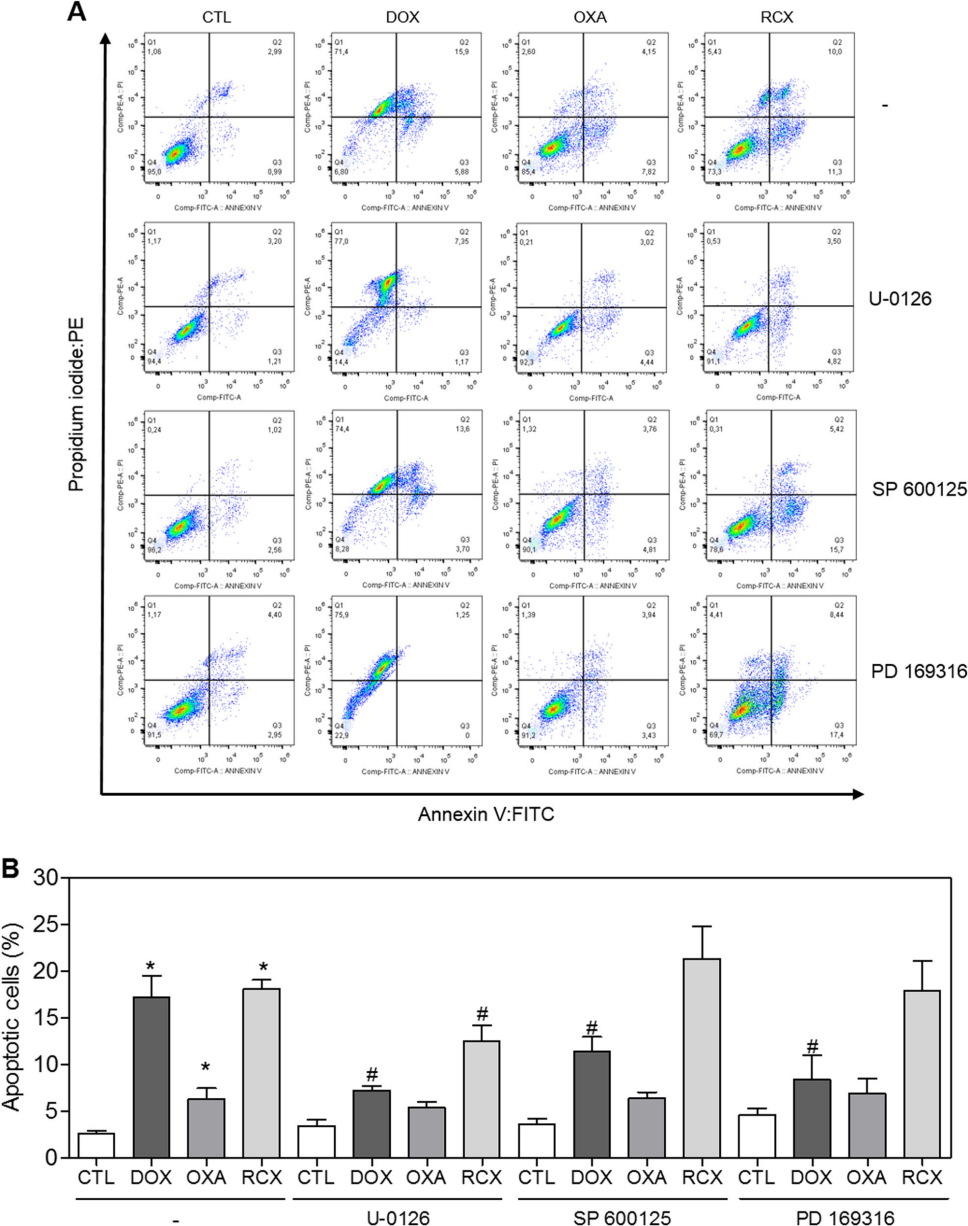
Effect of the MEK inhibitor (U-0126), JNK/SAPK inhibitor (SP 600125) and p38 MAPK inhibitor (PD 169316) on the apoptosis induced by the ruthenium complex with xanthoxylin (RCX) in HepG2 cells, as determined by flow cytometry using Annexin V-FITC/PI staining **a** Representative flow cytometric dot plots showing the percent of cells in the viable, early apoptotic, late apoptotic and necrotic stages. **b** Quantification of apoptotic HepG2 cells. For protection assays, the cells were pretreated for 2 h with 5 µM U-0126, 5 µM SP 600125 or 5 µM PD 169316 and then incubated with 12 µM RCX for 48 h. The negative control (CTL) was treated with the vehicle (0.2% DMSO) used for diluting the tested compound. Doxorubicin (DOX, 2 µM) and oxaliplatin (OXA, 10 µM) were used as positive controls. Data are presented as the means ± S.E.M. of three independent experiments performed in duplicate. Ten thousand events were evaluated per experiment, and cellular debris was omitted from the analysis. * *P* < 0.05 compared with the negative control by ANOVA, followed by the Student–Newman–Keuls test. # *P* < 0.05 compared with the respective treatment without inhibitor by ANOVA, followed by the Student–Newman–Keuls test

As observed above, the gene expression analysis indicated the activation of the MAPK pathway in RCX-treated HepG2 cells. Therefore, we decided to investigate the role of the three main MAPK families, extracellular signal-regulated kinase (ERK), Jun kinase (JNK/SAPK) and p38 MAPK, in RCX-induced apoptosis in HepG2 cells. For this, we monitored alterations in the phosphorylation status of ERK1/2, JNK/SAPK and p38 MAPK proteins by Phosflow analysis after acute (15 and 30 min) and prolonged (24 h) incubations with RCX (Fig. 8). RCX induced the phosphorylation of ERK1/2 after 15- and 30-min incubations and the phosphorylation of JNK/SAPK after a 15-min incubation, but an increase in the phosphorylation of p38 MAPK was not observed at any time point investigated. Moreover, pretreatment with an MEK (mitogen-activated protein kinase kinase) inhibitor (U-0126), which inhibits the activation of ERK1/2, prevented RCX-induced apoptosis (Figs. 9a, b). Pre-treatment with a JNK/SAPK inhibitor (SP 600125) or p38 MAPK inhibitor (PD 169316) did not prevent RCX-induced apoptosis (Figs. 9a, b). Since ERK1/2 activation is associated with the activation of the p53 pathway, we investigated the activation of the p53 pathway in RCX-treated HepG2 cells. However, pretreatment with a p53 inhibitor (cyclic pifithrin-α) did not prevent the RCX-induced apoptosis (Fig. S5A and S5B), indicating the activation of a p53-independent apoptosis pathway. In fact, the main gene of p53 pathway activation (TP53) was down-regulated, and the gene related to the inactivation of p53 (MDM2) was up-regulated in RCX-treated HepG2 cells.

### Ruthenium complex with xanthoxylin reduces HepG2 cell growth in a xenograft model

**Fig. 10:**
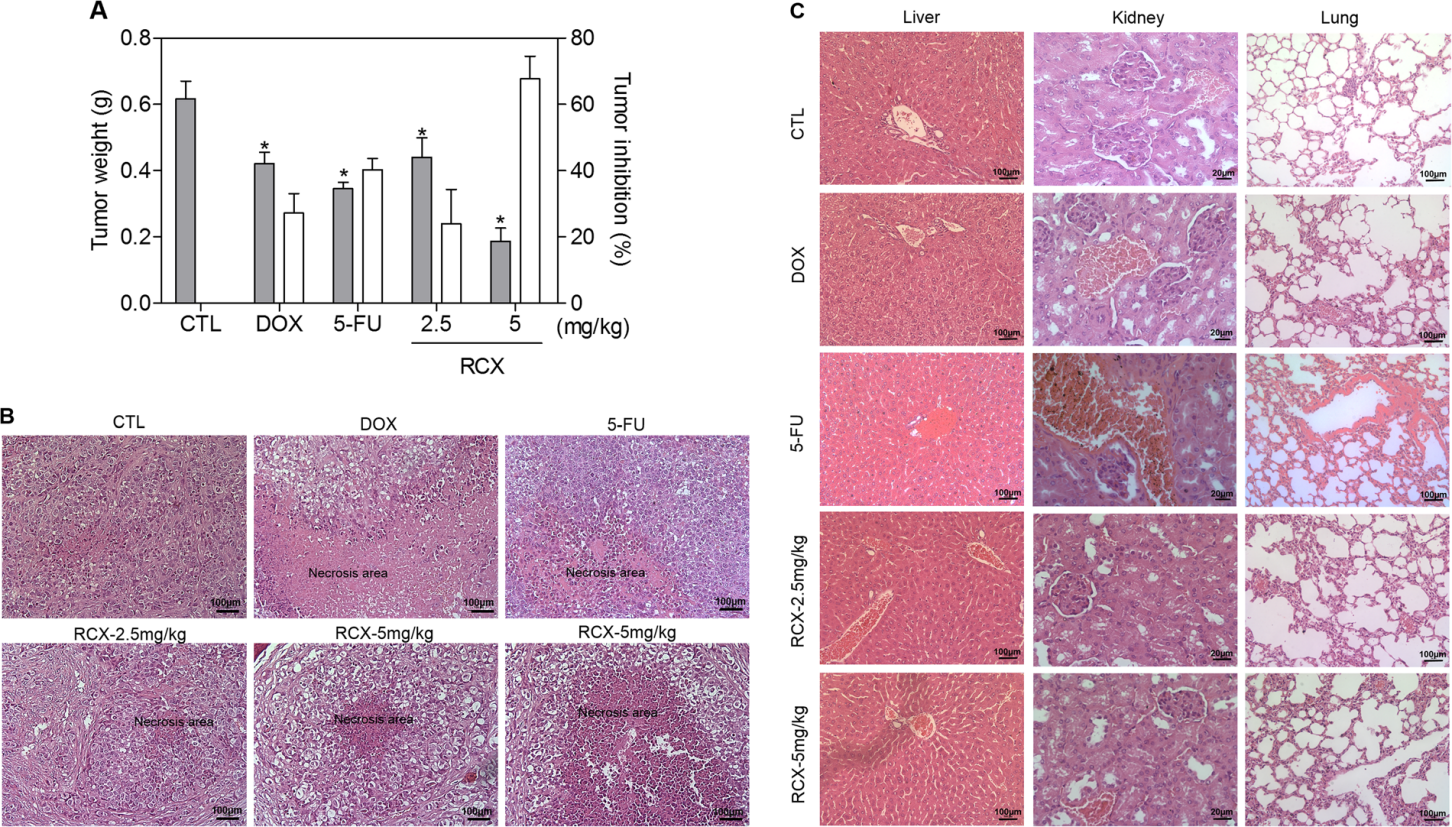
In vivo antitumor activity of the ruthenium complex with xanthoxylin (RCX) in C.B-17 SCID mice with HepG2 cell xenografts **a** Quantification of tumor weight and tumor inhibition. The gray bars represent tumor weight (*g*) and the white bars represent tumor inhibition (%). Data are presented as the means ± S.E.M. of 7–15 animals. * *P* < 0.05 compared with the negative control by ANOVA, followed by the Student–Newma–-Keuls test. **b** Representative histological analysis of the tumors stained with hematoxylin and eosin and analyzed by light microscopy. **c** Representative histological analyses of livers, kidneys and lungs stained with hematoxylin and eosin and analyzed by light microscopy. The negative control (CTL) was treated with the vehicle (5% DMSO) used for diluting the tested compound. Doxorubicin (DOX, 0.3 mg/kg) and 5-fluorouracil (5-FU, 10 mg/kg) were used as positive controls. Beginning 1 day after tumor implantation, the animals were treated through the intraperitoneal route for 21 consecutive days

The in vivo antitumor activity of RCX was investigated in C.B-17 severe combined immunodeficient (SCID) mice engrafted with HepG2 cells. The animals were treated with RCX at doses of 2.5 and 5 mg/kg by intraperitoneal injections once a day for 21 consecutive days. Both doses of RCX were able to inhibit HepG2 cell growth in mice. Figure 10a shows the inhibition of tumor growth. On the 22nd day, the average tumor weight of the negative control mice was 0.6 ± 0.04 g. In the presence of RCX, the average tumor weights were 0.4 ± 0.06 and 0.2 ± 0.04 g at the highest and lowest doses, respectively, and the tumor mass inhibition was 24.0 and 67.7%, respectively. The positive controls (0.3 mg/kg doxorubicin and 10 mg/kg 5-fluorouracil) reduced the tumor weight by 27.2 and 40.3%, respectively. In the histological analysis, all groups exhibited solid, hypervascularized tumors with cells exhibiting intense pleomorphism and prominent nucleoli. Necrotic areas were observed in all groups, but aberrant mitotic figures were less evident in the RCX-treated groups (Fig. 10b).

Toxicological parameters were examined in the RCX-treated mice. Compared with the weight of the negative control mice, a slight decrease in the body weight of C.B-17 SCID mice bearing HepG2 cells was identified after 21 days of treatment with RCX at the highest dose (*P* < 0.05); however, there was no change in the body weight of animals treated with RCX at the lowest dose (*P* > 0.05). No significant alterations were observed in the liver, kidney, lung or heart wet weight of any group (*P > *0.05) (Table S3). The number of white and erythrocyte blood cells in the RCX-treated animals was also analyzed (Table S4). The number of leukocytes remained unchanged after treatment with both doses of RCX (*P* > 0.05); however, we found a decrease in the total number of leukocytes in the doxorubicin and 5-fluorouracil groups compared to that of the negative control group (*P < *0.05). No significant alteration in the number of erythrocytes was observed in any group.

Morphological analyses of the liver, kidneys, lungs and hearts in all groups were performed (Fig. 10c). Histopathological analysis of the livers revealed hydropic degeneration, portal venous system dilatation, bile duct dilatation with areas of atrophy, disperse areas of coagulation necrosis and inflammation in all experimental groups. It is important to note that these histopathological characteristics were more pronounced in the RCX groups than in the other groups (negative control, 5-fluorouracil and doxorubicin). In the kidneys, mild vascular congestion and moderate glomerular hyalinization were observed in all groups. In the lungs of all animals, atelectasis, focal hemorrhage, acute inflammation, vascular congestion, thickening of the alveolar septa and increased airspace were observed. It should be noted that these histopathological characteristics were more pronounced in the doxorubicin and RCX (both doses) groups than in the other groups (negative control and 5-fluorouracil). In addition, nodules and tumor emboli were observed in the pulmonary parenchyma of two animals from the negative control group. Histopathological analysis of animal hearts did not show alterations in any group. Some histopathological features of this study (hydropic degeneration, vascular congestion and focal areas of inflammation) are acute cellular responses to stimuli unrelated to the treatment, and the injured cells are able to return to a homoeostatic state when the stimulation ends.

## Discussion

In this study, a novel ruthenium complex with xanthoxylin was synthesized and assessed for its cellular and molecular response in human hepatocellular carcinoma HepG2 cells for the first time. As mentioned, ruthenium complexes have been extensively reported as potent cytotoxic compounds in different cancer cells;^6–9^ however, the structure of the ligand confers the characteristic properties of the metal complex formed. Xanthoxylin is a non-cytotoxic compound that forms a ruthenium complex with potent cytotoxicity in cancer cells.

EDS analysis of RCX-treated HepG2 cells indicated the accumulation of ruthenium in the cell nucleus. Moreover, RCX induced DNA intercalation, inhibited DNA synthesis and triggered the caspase-mediated apoptosis pathway in HepG2 cells, as observed by cell shrinkage, internucleosomal DNA fragmentation, externalization of phosphatidylserine, loss of mitochondrial transmembrane potential and activation of caspase-3, -8 and -9. In addition, the apoptosis induced by RCX was prevented by pretreatment with a pan-caspase inhibitor, a caspase-8 inhibitor and a caspase-9 inhibitor. Indeed, ruthenium complexes with different ligands have been detected to have DNA-binding ability in cell nuclei, leading to the inhibition of DNA synthesis and the induction of apoptosis through the death receptor, mitochondria and/or oxidative stress pathways^6–9,21–23^.

RNA transcript analysis revealed changes in genes related to cell cycle control, apoptosis and the MAPK pathway. Of the genes related to cell cycle control, one is required for cell cycle G_1_/S transition (CDKN2A), and the genes related to apoptosis included the pro-apoptotic genes (BCL2L11, FAS and E2F1). In fact, S-phase arrest was observed in RCX-treated HepG2 cells, followed by the induction of the caspase-mediated apoptosis pathway. Jovanović et al^24^. reported that a ruthenium-arene complex with an isoquinoline-3-carboxylic acid ligand caused S-phase arrest and cell death through the intrinsic (mitochondrial) apoptotic pathway, which was mediated by ROS. The ruthenium complex [Ru(dmp)_2_(NMIP)](ClO_4_)_2_ also arrested cell growth at the S-phase and led to cell death by apoptosis in osteosarcoma cells^25^.

In regards to the role of MAPK signaling in RCX-treated HepG2 cells, RCX induced the phosphorylation of ERK1/2. In addition, pretreatment with an MEK inhibitor (U-0126) prevented the RCX-induced apoptosis, indicating the activation of ERK1/2-mediated apoptosis in HepG2 cells. Although ERK1/2 has a pro-survival function in the MAPK signaling pathway, ERK1/2 activation can also promote apoptosis. Etoposide, platinum compounds and other DNA-damaging agents activate ERK1/2 in different cell lines, and the inhibition of ERK1/2 activation attenuates the apoptosis induced by these molecules^26–29^. ERK1/2-mediated apoptosis caused by DNA-damaging agents can occur by direct DNA damage or via ROS production. We found here that RCX does not target oxidative stress but induces DNA intercalation. Additionally, the p53 pathway is also activated during cell DNA damage. In contrast, although there are links between ERK and p53 activation during DNA damage-induced stress, ERK activation can induce apoptosis by either a p53-dependent or p53-independent pathway^30,31^. Interestingly, pretreatment with a p53 inhibitor (cyclic pifithrin-α) did not prevent RCX-induced apoptosis, indicating the activation of ERK1/2-mediated apoptosis in HepG2 cells by the p53-independent pathway.

RCX also reduced HepG2 cell growth in the xenograft model more efficiently than doxorubicin and 5-fluorouracil and reduced the number of mitotic figures in HepG2 cells. Deng et al^23^. demonstrated that the ruthenium complex with the phenylterpyridine derivative inhibited in vivo A375 tumor development in a xenograft tumor model. The ruthenium imidazole complex also significantly inhibited tumor growth in mice bearing A549 xenografts^32^.

**Fig. 11:**
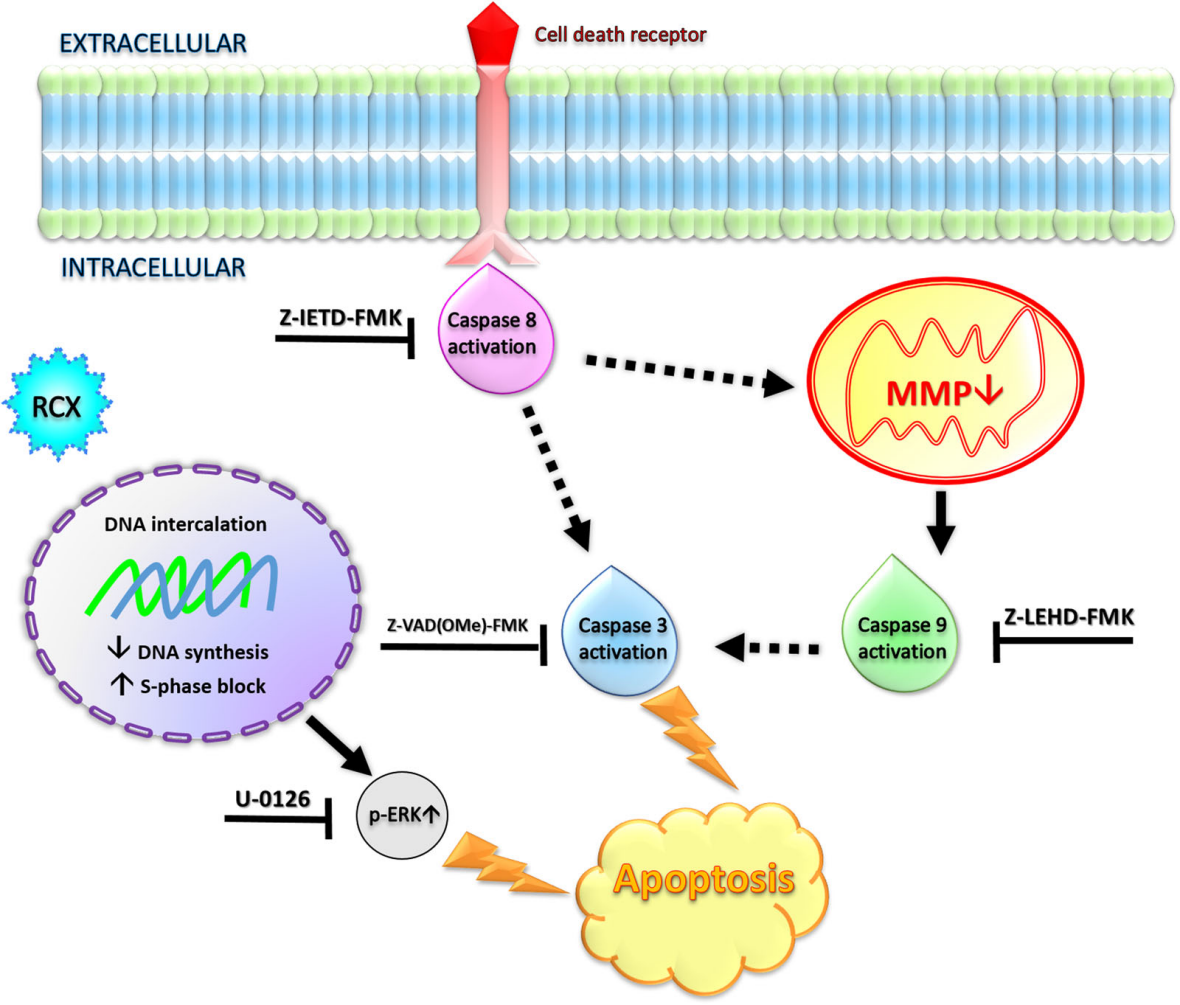
Summary of the molecular mechanisms of the novel ruthenium complex with xanthoxylin (RCX) in HepG2 cells

In summary, our study revealed that RCX exhibits potent cytotoxicity in a panel of different cancer cells, which was associated with DNA intercalation and the inhibition of DNA synthesis. We observed S-phase arrest, followed by the induction of caspase-mediated and ERK1/2-mediated apoptosis in HepG2 cells by a p53-independent pathway (Fig. 11). Moreover, RCX reduced HepG2 cell growth in the xenograft model, indicating that RCX is a novel anticancer drug candidate.

## Materials and methods

### Synthesis of novel ruthenium complex with xanthoxylin

The RuCl_3_3H_2_O, lithium chloride (LiCl), 1,10′-phenanthroline (phen) and xanthoxylin (xant) were all purchased from Sigma-Aldrich (St. Louis, MO, USA) and were used to synthesize RCX with the chemical formula *cis*-[Ru(phen)_2_(xant)](PF_6_). The precursor *cis*-RuCl_2_(phen)_2_2H_2_O was obtained as dark violet crystals following the procedure previously described^33^. Xanthoxylin (0.0549 g, 0.28 mmol) and triethylamine (39 mL, 0.28 mmol) were dissolved in a 1:1 EtOH/H_2_O mixture (30 mL), and 0.15 g (0.26 mmol) of *cis*-[RuCl_2_(phen)_2_], was added. The solution was stirred under an N_2_ atmosphere for 8 h under reflux. A stoichiometric amount of NH_4_PF_6_ was added to precipitate the complex, and the resulting mixture was cooled at 0 °C overnight. The *cis*-[Ru(phen)_2_(xant)](PF_6_) was filtered, washed with cool water, ethanol and diethyl ether and dried under vacuum (80% yield).

^1^H NMR spectra were measured in a DMSO-*d*_6_ solution using a Bruker DRX-400 or DRX-500 spectrometer (Bruker Biospin Gmbh, Rheinstetten, Germany). All chemical shifts (δ) are given in ppm with reference to the hydrogen signal of the methyl group of tetramethylsilane as the internal standard, and the coupling constants (J) are in Hz. Electrochemical measurements were recorded using a μAutolab Type III (Eco-Chemie B.V., Utrecht, The Netherlands) potentiostat. Dimethylformamide solutions typically contained 1.0 × 10^−3^ mol L^−1^ of the ruthenium complex. A platinum disk served as the working (*d* = 0.2 mm) and counter (*d* = 0.5 mm) electrode, and Ag^+^/Ag wire was used as the reference electrode. Solutions contained 0.1 M tetra-n-butylammonium hexafluorophosphate (TBAPF_6_) as the supporting electrolyte. Optical spectra were recorded on an Agilent 8453 UV-vis spectrophotometer (Agilent Technologies, Waldbronn, Germany). IR spectra were recorded on an FT-IR Bomem Michelson FT spectrometer in the 4000–400 cm^−1^ region.

### In vitro assays

#### Cells

A panel of 15 cancer cell lines, 3 non-cancer cells and 1 mutant and its parental cell line were selected for this study, as detailed in Table S5. The cells were cultured in RPMI 1640 medium (Gibco-BRL, Gaithersburg, MD, USA) with 10% fetal bovine serum (Life, Carlsbad, CA, USA), 2 mM L-glutamine (Vetec Química Fina, Duque de Caxias, RJ, Brazil) and 50 μg/mL gentamycin (Life, Carlsbad, CA, USA). Adherent cells were collected by treatment with 0.25% trypsin EDTA solution (Gibco-BRL). All cell lines were cultured in flasks at 37 °C in 5% CO_2_ and subcultured every 3–4 days to maintain exponential growth. All cell lines were tested for mycoplasma using a mycoplasma stain kit (Sigma-Aldrich) to validate the use of cells free from contamination. Heparinized blood was collected from 20- to 35-year-old, non-smoker healthy donors who had not taken any drugs for at least 15 days prior to sample collection, and peripheral blood mononuclear cells (PBMCs) were isolated using a Ficoll density gradient with GE Ficoll-Paque Plus reagent (GE Healthcare Bio-Sciences AB, Sweden). PBMCs were washed and resuspended at a concentration of 3 × 10^5^ cells/mL in RPMI 1640 medium with 20% fetal bovine serum, 2 mM glutamine and 50 μg/mL gentamycin at 37 °C with 5% CO_2_. Concanavalin A (ConA, Sigma-Aldrich) was used as a mitogen to trigger cell division in T-lymphocytes. ConA (10 μg/mL) was added at the beginning of culture, and the cells were treated with the complex and tested after 24 h. Cell viability in all experiments was examined using the TBE assay. Over 90% of the cells were viable at the beginning of the culture. The Research Ethics Committee of the Oswaldo Cruz Foundation (Salvador, Bahia, Brazil) approved the experimental protocol (# 031019/2013). All participants signed a written informed consent to participate in the study.

#### Cytotoxic activity assay

Cell viability was quantified colorimetrically using the alamar blue assay according to Ahmed et al.^34^. Briefly, cells were inserted in 96-well plates for all experiments (7 × 10^4^ cells/mL or 3 × 10^5^ cells/mL for adherent and non-adherent cells, respectively, in 100 μL of medium) and incubated at 37 °C in 5% CO_2_ atmosphere overnight. Then, RCX was dissolved in 0.5% DMSO at a range of eight different concentrations from 0.19 to 25 μg/mL, was added to each well and incubated for 72 h. Negative controls were treated with the vehicle that was used for diluting the tested compound. Doxorubicin (purity ≥ 95%, doxorubicin hydrochloride, Laboratory IMA S.A.I.C., Buenos Aires, Argentina) and oxaliplatin (Sigma-Aldrich) were used as the positive controls. Four (for cell lines) or twenty-four hours (for PBMCs) before the end of incubation, 20 μL of alamar blue (resazurin, Sigma-Aldrich) stock solution (0.312 mg/mL) was added to each well. Absorbance was measured at 570 nm and 600 nm using the SpectraMax 190 Microplate Reader (Molecular Devices, Sunnyvale, CA, USA), and the drug effect was quantified as a percent of the absorbance of the control-treated cells.

#### 3D multicellular spheroid culture

HepG2 cells were cultivated in 3D multicellular spheroids. Briefly, 100 μL of cell solution (0.5 × 10^6^ cells/mL) was added to a 96-well plate with a cell-repellent surface (Greiner Bio-One, Kremsmünster, Austria) and cultured in complete medium plus 3% Matrigel (BD Biosciences, San Jose, CA, USA). Spheroids with stable structures formed after three days. Then, the spheroids were exposed to a range of drug concentrations for 72 h. The negative control received the vehicle that was used for diluting the complex tested. In the end of the experiment, morphological changes were examined by light microscopy (Olympus BX41, Tokyo, Japan) using Image-Pro software (Media Cybernetics, Inc. Silver Spring, USA), and cell viability was quantified by alamar blue assay as described above.

#### Trypan blue exclusion assay

The TBE assay was used to confirm the cytotoxic effect of the complex tested. The number of viable cells and non-viable (take up trypan blue) cells were counted. Briefly, 90 μL was removed from the cell suspension and 10 μL of trypan blue (0.4%) was added. Cell counting was performed using a light microscope with a hemocytometer filled with an aliquot of the homogenized cell suspension.

#### Ruthenium subcellular distribution

The analysis of the subcellular distribution of ruthenium in HepG2 cells was performed by EDS^35^. Briefly, cells were fixed in 0.1 M sodium cacodylate buffer (pH 7.4) containing 2.5% glutaraldehyde and 2% paraformaldehyde for at least 2 h. After washing, the cells were dehydrated in an acetone series and embedded in Polybed epoxy resin (Polysciences; Warrington, PA). Ultrathin sections were examined under a JEM-1230 TEM integrated with an EDS microanalytics system (JEOL USA, Inc., Peabody, MA, USA).

#### DNA intercalation assay

DNA intercalation was assessed by examining the ability of the complex to displace ethidium bromide from ctDNA (Sigma-Aldrich)^36^. The assay was conducted in 96-well plates, and the reaction mixture contained 15 µg/mL ctDNA, 1.5 µM ethidium bromide and either 10 or 20 µM RCX in 100 µL of saline solution. After a 15 min of incubation at room temperature, fluorescence was measured using the excitation and emission wavelengths of 320 nm and 600 nm, respectively, using the Spectramax Microplate Reader (Molecular Devices).

#### DNA synthesis quantification

Cellular DNA synthesis was quantified by the incorporation of methyl-[^3^H]-thymidine. Methyl-[^3^H]-thymidine is a radiolabeled DNA precursor that is incorporated into newly synthesized DNA. Briefly, the complex was added along with 1 µCi of methyl-[^3^H]-thymidine (PerkinElmer; Shelton, CT, USA) and incubated for 3 h. After this incubation period, cultures were harvested using a cell harvester (Brandel, Inc., Gaithersburg, MD, USA), and the amount of incorporated^3^H-thymidine was determined using a liquid scintillation cocktail Hidex Maxilight (PerkinElmer Life Sciences; Groningen, GE, Netherlands) and a plate CHAMELEON V multilabel Counter (Mustionkatu 2, Turku, Finland). Mitoxantrone (Sigma-Aldrich) was used as the positive control.

#### Internucleosomal DNA fragmentation and cell cycle distribution

Internucleosomal DNA fragmentation and cell cycle distribution analyses followed the procedure previously described^37^. Briefly, the cells were harvested in a permeabilization solution containing 0.1% Triton X-100, 2 µg/mL PI, 0.1% sodium citrate and 100 µg/mL RNAse (all from Sigma-Aldrich) and incubated in the dark for 15 min at room temperature. Finally, cell fluorescence was measured by flow cytometry with a BD LSRFortessa cytometer using BD FACSDiva Software (BD Biosciences) and FlowJo Software 10 (FlowJo Lcc; Ashland, OR, USA). Ten thousand events were evaluated per experiment, and cellular debris was omitted from the analysis.

#### Morphological analysis

To evaluate alterations in morphology, cells were cultured on a coverslip and stained with May-Grunwald-Giemsa. Morphological changes were examined by light microscopy (Olympus BX41) using Image-Pro software (Media Cybernetics). Light scattering features were determined by flow cytometry, as described above. In addition, cells were fixed in 0.1 M sodium cacodylate buffer (pH 7.4) containing 2.5% glutaraldehyde and 2% paraformaldehyde for at least 2 h. After washing, the cells were treated with 1% osmium tetroxide, 0.8% potassium ferricyanide and 5 mM calcium chloride for 1 h. After another washing, the cells were dehydrated in acetone series and embedded in Polybed epoxy resin. Ultrathin sections were stained with 2% aqueous uranyl acetate and 2% aqueous lead citrate, and the ultrastructure analysis was performed by TEM using a JEM-1230 microscope (JEOL USA, Inc.).

#### Annexin V/PI staining assay

For apoptosis detection, we used the FITC Annexin V Apoptosis Detection Kit I (BD Biosciences), and the analysis was performed according to the manufacturer’s instructions. Briefly, cells were washed twice with saline and then resuspended in 100 μL of binding buffer with 5 μL of PI and 5 μL of FITC Annexin V. The cells were gently mixed by vortexing and incubated for 15 min at room temperature in the dark. Afterwards, 400 μL of binding buffer was added to each tube, and the cell fluorescence was determined by flow cytometry, as described above. The percent of viable, early apoptotic, late apoptotic and necrotic cells were determined. Protection assays using the pan-caspase inhibitor (Z-VAD(Ome)-FMK, Cayman Chemical; Ann Arbor, MI, USA), caspase-8 inhibitor (Z-IETD-FMK; BD Biosciences), caspase-9 inhibitor (Z-LEHD-FMK; BD Biosciences), MEK inhibitor (U-0126; Cayman Chemical), JNK/SAPK inhibitor (SP 600125; Cayman Chemical), p38 MAPK inhibitor (PD 169316; Cayman Chemical) and p53 inhibitor (cyclic pifithrin-α; Cayman Chemical) were also performed. In brief, the cells were pretreated for 2 h with 50 µM Z-VAD(Ome)-FMK, 20 µM Z-IETD-FMK, 20 µM Z-LEHD-FMK, 5 µM U-0126, 5 µM SP 600125, 5 µM PD 169316 or 10 µM cyclic pifithrin-α, followed by incubation with 12 µM RCX for 48 h. The cells were then trypsinized, and the FITC Annexin V Apoptosis Detection assay was conducted as described above.

#### Measurement of the mitochondrial transmembrane potential

Mitochondrial transmembrane potential was determined by the retention of the rhodamine 123 dye^38^. Cells were incubated with rhodamine 123 (5 μg/mL, Sigma-Aldrich Co.) at 37 °C for 15 min in the dark and washed with saline. The cells were then incubated again in saline at 37 °C for 30 min in the dark, and the cell fluorescence was determined by flow cytometry, as described above.

#### Caspase-3, -8 and -9 activation assays

A caspase-3 colorimetric assay kit (Sigma-Aldrich), caspase-8 colorimetric assay kit (BioVision Inc.; Milpitas, CA, USA) and caspase-9 colorimetric assay kit (Invitrogen; Frederick, MD, USA) were used to investigate the activation of caspase-3, -8 and -9 in RCX-treated HepG2, respectively. The analysis was performed according to the manufacturer’s instructions. Briefly, cells were lysed by incubation with cell lysis buffer on ice for 10 min and then centrifuged. Enzyme reactions were carried out in a 96-well flat-bottom microplate. To each reaction mixture, 5 μL of cell lysate was added. Absorbance at 405 nm was measured using the SpectraMax 190 Microplate Reader (Molecular Devices). The results are expressed as the specific activity (IU/mg protein) of each caspase.

#### Measurement of intracellular reactive oxygen species levels

The production levels of ROS were measured according to a previously described method^39^ using DCF-DA (Sigma-Aldrich). In brief, cells were treated with RCX for 1 or 3 h. Then, the cells were collected, washed with saline and resuspended in FACS tubes with saline containing 5 μM DCF-DA for 30 min. Finally, the cells were washed with saline, and cell fluorescence was determined by flow cytometry as described above. A protection assay using the antioxidant NAC (Sigma-Aldrich) was also performed. In brief, the cells were pretreated for 1 h with 5 mM NAC and then incubated with 12 µM RCX for 24 h. The cells were then trypsinized, and the TBE assay was performed.

#### Gene expression analysis by qPCR array

HepG2 cells were plated in bottles for cell culture (7 × 10^4^ cells/mL). After 12 h of incubation with 12 µM RCX, total RNA was isolated from the cells using an RNeasy Plus Mini Kit (Qiagen; Hilden, Germany) according to the manufacturer’s instructions. The RNA was evaluated by fluorimetry using QuBit (Life Technologies; Camarillo, CA, USA). RNA reverse transcription was performed using a Superscript VILO™ Kit (Invitrogen Corporation; Waltham, MA, USA). A 96-well plate TaqMan® Array Human Molecular Mechanisms of Cancer (ID 4418806, Applied Biosystems^TM^, Foster City, CA, USA) was used for the gene expression study by qPCR. The reactions were conducted in an ABI ViiA7 system (Applied Biosystems^TM^; Foster City, CA, USA). The cycle conditions comprised 2 min at 50 °C, 20 s at 95 °C, then 40 cycles of 3 s at 95 °C and 30 s at 60 °C. The relative quantification (RQ) of mRNA expression was calculated by the 2^-ΔΔCT^ method^40^ using Gene Expression Suite™ Software (Applied Biosystems^TM^), and the cells treated with the negative control (0.2% DMSO) were used as a calibrator. The reactions were normalized by the geometric mean of the RQ of the reference genes GAPDH, 18S and HPRT1. All experiments were performed in DNase/RNase-free conditions. The genes were considered to be up-regulated if the RQ ≥ 2, which means that the gene expression in RCX-treated cells was at least twice that of the negative control-treated cells. Similarly, the genes were considered to be down-regulated if RQ ≤ 0.5, which means that the gene expression in RCX-treated cells was at least half of that of the negative control-treated cells.

#### Phosflow analysis

Phosphorylated ERK 1/2, JNK/SAPK and p38 MAPK were analyzed by flow cytometry^41^. In brief, cells were collected and resuspended in 0.5–1 mL of 4% formaldehyde and fixed for 10 min at 37 °C. Then, the tubes were chilled on ice for 1 min. The cells were permeabilized by slowly adding ice-cold 100% methanol to prechilled cells while gently vortexing to a final concentration of 90% methanol and were incubated for 30 min on ice. After washing with incubation buffer (0.5% bovine serum albumin in PBS), PE mouse anti-ERK1/2 (pT202/pY204), PE mouse anti-JNK/SAPK (pT183/pY185), PE mouse anti-p38 MAPK (pT180/pY182) or PE mouse IgG_1_, κ isotype control antibodies, all from BD Biosciences, were added and incubated for 1 h at room temperature. Finally, the cells were washed with PBS, and cell fluorescence was determined by flow cytometry as described above.

### In vivo assays

#### Animals

A total of 78 C.B*-*17 SCID mice (females, 25–30 g) was obtained and maintained at the animal facilities from Gonçalo Moniz Institute-FIOCRUZ (Salvador, Bahia, Brazil). Animals were housed in cages with free access to food and water. All animals were kept under a 12:12 h light-dark cycle (lights on at 6:00 a.m.). The local Animal Ethics Committee approved the experimental protocol (number 06/2015).

#### Human hepatocellular carcinoma xenograft model

HepG2 cells (1 × 10^7^ cells per 500 µL) were implanted subcutaneously into the left front armpit of the mice. At the beginning of the experiment, mice were randomly divided into five groups: group 1 animals received injections of vehicle with 5% DMSO solution (*n* = 15); group 2 animals received injections of doxorubicin (0.3 mg/kg, *n* = 14); group 3 animals received injections of 5-fluorouracil (10 mg/kg, *n* = 15, Sigma-Aldrich); group 4 animals received injections of RCX at 2.5 mg/kg (*n* = 14); and group 5 animals received injections of RCX at 5 mg/kg (*n* = 14). The treatments were initiated one day after the tumor cell injection. The animals were treated intraperitoneally (200 µL per animal) once daily for 21 consecutive days. On the 22nd day, the animals were anesthetized, and peripheral blood samples were collected from the brachial artery. Animals were euthanized by anesthetic overdose, and tumors were excised and weighed.

#### Toxicological evolution

To assess the toxicological effects, mice were weighed at the beginning and at the end of the experiment. Animals were observed for signs of abnormalities throughout the study. Hematological analysis was performed using light microscopy. Livers, kidneys, lungs and hearts were removed, weighed and examined for any signs of gross lesions, color changes and/or hemorrhages. After gross macroscopic examination, the tumors, livers, kidneys, lungs and hearts were fixed in 4% formalin buffer and embedded in paraffin. Tissue sections were stained with hematoxylin and eosin chromogens, and a pathologist performed the analysis under a light microscope.

### Statistical analysis

Data are presented as the means ± S.E.M. or as IC_50_ values with 95% confidence intervals (CI 95%) obtained by nonlinear regression. Differences among experimental groups were compared using analysis of variance (ANOVA) followed by the Student–Newman–Keuls test (*P* < 0.05). All statistical analyses were performed using GraphPad Prism (Intuitive Software for Science; San Diego, CA, USA).

## Electronic supplementary material

Electronic supplementary material
