# Supplementary material for: A novel ruthenium complex with xanthoxylin induces S-phase arrest and causes ERK1/2-mediated apoptosis in HepG2 cells through a p53-independent pathway
Source: Cell Death Dis. 2018 Jan 23;9(2):79. doi: 10.1038/s41419-017-0104-6 (PMC5833756; doi:10.1038/s41419-017-0104-6)
Supplement: Supplementary file 1 — Electronic supplementary material [file 41419_2017_104_MOESM1_ESM.doc]

**Supplementary information**

**A novel ruthenium complex with xanthoxylin induces S-phase arrest and causes ERK1/2-mediated apoptosis in HepG2 cells through a p53-independent pathway**

Nanashara C. de Carvalho^1^, Sara P. Neves^1^, Rosane B. Dias^1^, Ludmila de F. Valverde^1^, Caroline B. S. Sales^2^, Clarissa A. G. Rocha^1^, Milena B. P. Soares^1,3^, Edjane R. dos Santos^4^, Regina M. M. Oliveira^4^, Rose M. Carlos^4^, **Paulo C. L. Nogueira^5^,** Daniel P. Bezerra^1^,*

^1^Gonçalo Moniz Institute, Oswaldo Cruz Foundation (IGM-FIOCRUZ/BA), Salvador, Bahia, 40296-710, Brazil.

^2^Department of Biomorphology, Institute of Health Sciences, Federal University of Bahia , Salvador, Bahia, 40110-902, Brazil.

^3^Center of Biotechnology and Cell therapy, Hospital São Rafael, Salvador, Bahia, 41253-190, Brazil.

^4^Department of Chemistry, Federal University of São Carlos, São Carlos, São Paulo, 13561-901, Brazil.

^5^Department of Chemistry, Federal University of Sergipe, São Cristóvão, Sergipe, 49100-000, Brazil.

*** Corresponding author**

Prof. Dr. Daniel P. Bezerra, Gonçalo Moniz Institute, Oswaldo Cruz Foundation (IGM-FIOCRUZ/BA), Rua Waldemar Falcão, 121, Candeal, 40296-710, Salvador, Bahia, Brazil. E-mail: danielpbezerra@gmail.com Tel/Fax + 55 71

**Figure S1.** Effect of the ruthenium complex with xanthoxylin (RCX) in the cell viability of HepG2 cells, as determined by the trypan blue staining after 12 (**A**), 24 (**B**), 48 (**C**) and 72 (**D**) h of incubation. The gray bars represent the viable cells and the white bars represent the non-viable cells. The negative control (CTL) was treated with the vehicle (0.2% DMSO) used for diluting the tested compound. Doxorubicin (DOX, 2 µM) and oxaliplatin (OXA, 10 µM) were used as positive controls. Data are presented as the means ± S.E.M. of three independent experiments performed in duplicate. * *P* < 0.05 compared with the negative control by ANOVA, followed by the Student Newman-Keuls Test.

**
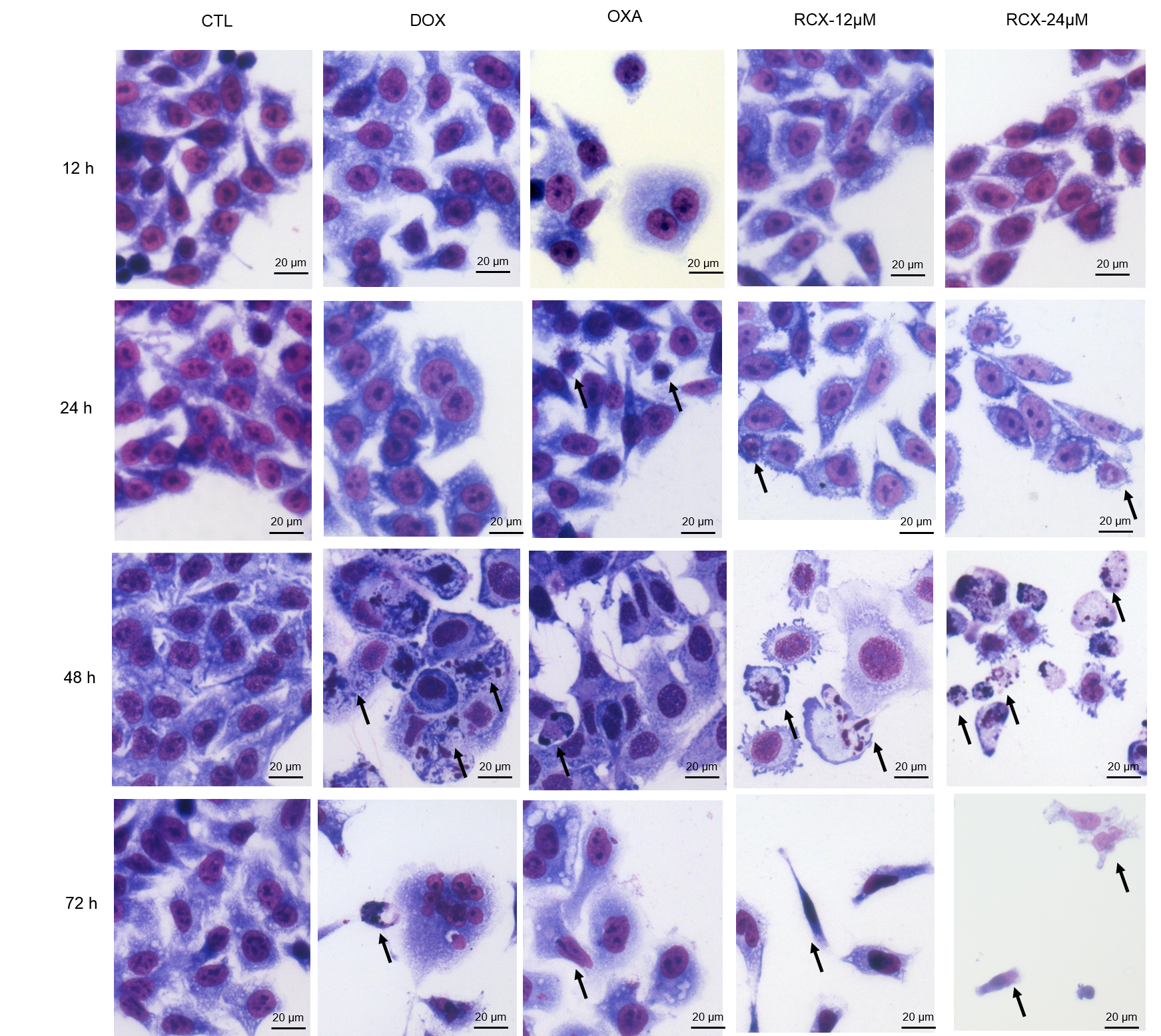
**

**Figure S2.** Effect of ruthenium complex with xanthoxylin (RCX) in the morphological analysis of HepG2 cells after 12, 24, 48 and 72 h of incubation. The cells were stained with may-grunwald-giemsa and examined by light microscopy (bar = 20 µm). Arrows indicate cell shrinkage or cells with fragmented DNA. The negative control (CTL) was treated with the vehicle (0.2% DMSO) used for diluting the tested compound. Doxorubicin (DOX, 2 µM) and oxaliplatin (OXA, 10 µM) were used as positive controls.


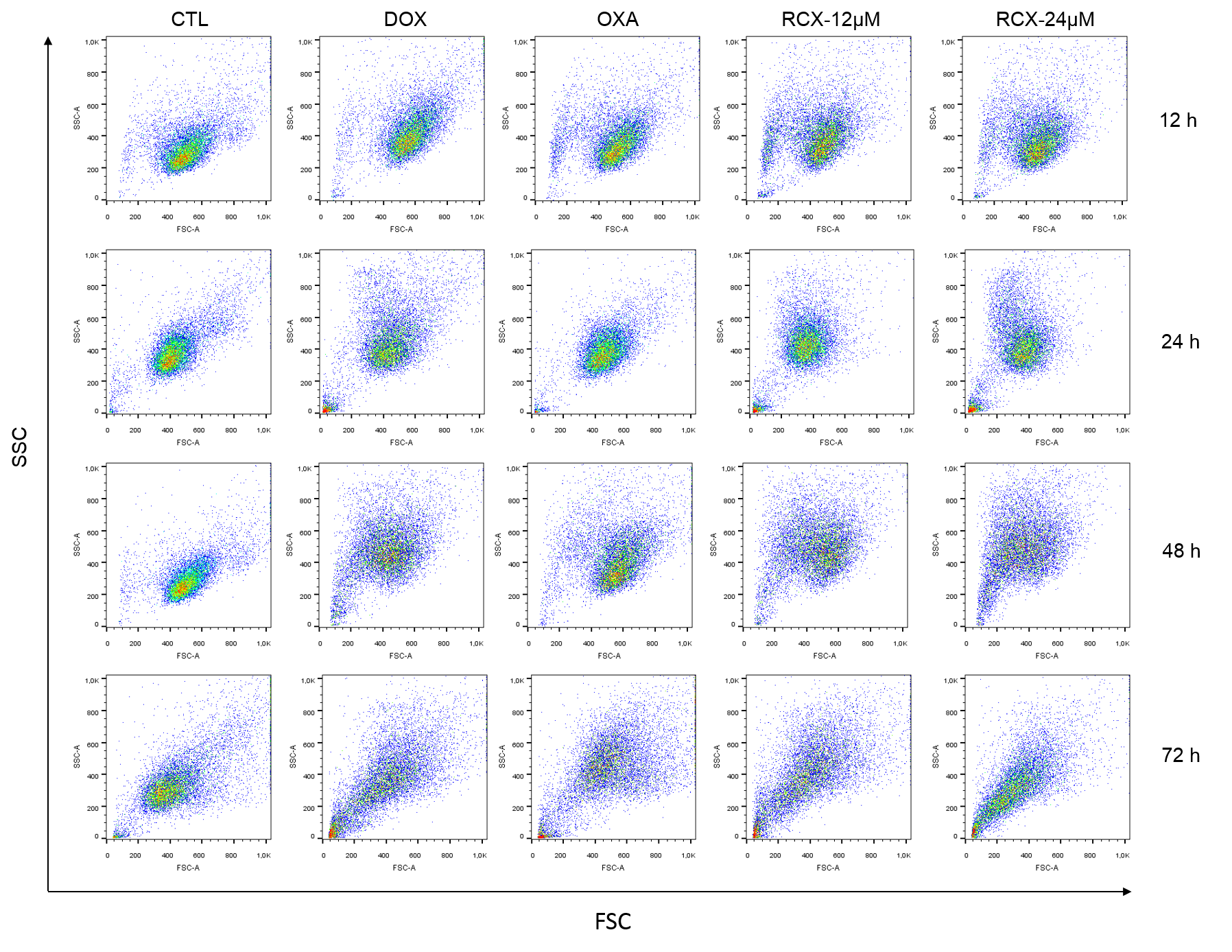


**Figure S3.** Effect of ruthenium complex with xanthoxylin (RCX) in the morphological analysis of HepG2 cells after 12, 24, 48 and 72 h of incubation. Light scattering features were determined by flow cytometry. Ten thousands events were evaluated per experiment and cellular debris was omitted from the analysis. The negative control (CTL) was treated with the vehicle (0.2% DMSO) used for diluting the tested compound. Doxorubicin (DOX, 2 µM) and oxaliplatin (OXA, 10 µM) were used as positive controls. FSC: forward light scatter. SCC: side scatter.

**Figure S4.** Effect of ruthenium complex with xanthoxylin (RCX) in the levels of reactive oxygen species (ROS) of HepG2 cells and protection by NAC, as determined by flow cytometry using DCF-DA staining. (**A**) ROS levels of HepG2 cells after 1 and 3 h incubation. (**B**) Cellular viability of HepG2 cells pre-treated with the antioxidant NAC and, then treated with RCX, as determined by the trypan blue staining. For the protection assay, the cells were pre-treated for 1 h with 5 mM NAC and then incubated with 12 µM RCX for 24 h. The negative control (CTL) was treated with the vehicle (0.2% DMSO) used for diluting the tested compound. Hydrogen peroxide (H_2_O_2_, 200 µM), doxorubicin (DOX, 2 µM) and oxaliplatin (OXA, 10 µM) were used as positive controls. Data are presented as the means ± S.E.M. of three independent experiments performed in duplicate or triplicate. For flow cytometry analysis, 10,000 events were evaluated per experiment and cellular debris was omitted from the analysis. * *P* < 0.05 compared with the negative control by ANOVA, followed by the Student Newman-Keuls Test. # *P* < 0.05 compared with the respective treatment without inhibitor by ANOVA, followed by the Student Newman-Keuls Test.

**
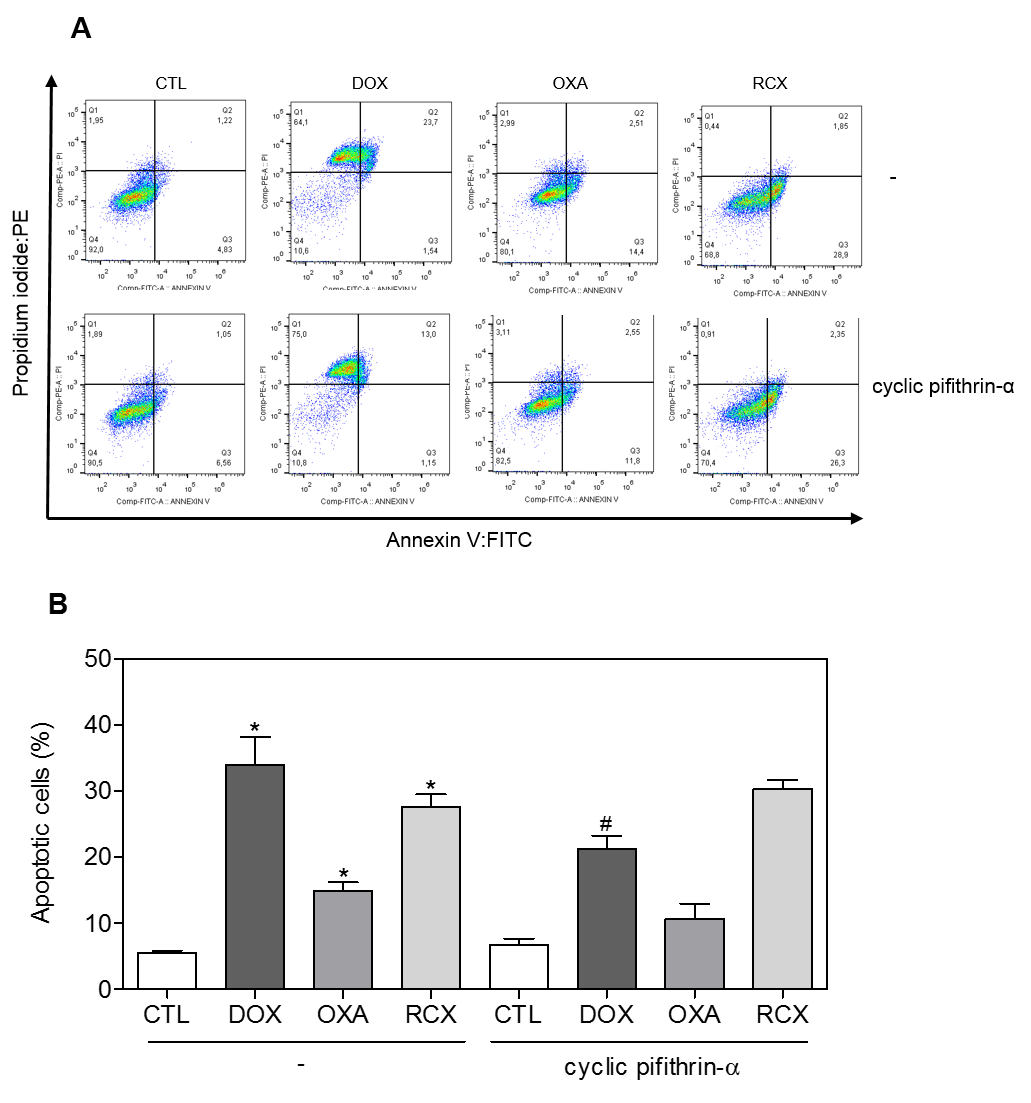
**

### Figure S5. Effect of the p53 inhibitor (**cyclic pifithrin-α**) in the apoptosis induced by the ruthenium complex with xanthoxylin (RCX) in HepG2 cells, as determined by flow cytometry using Annexin V-FITC/PI staining. (A) Representative flow cytometry dot plots show the percent of cells in the viable, early apoptotic, late apoptotic and necrotic stage. (B) Quantification of apoptotic HepG2 cells. The cells were pretreated for 2 h with 10 µM **cyclic pifithrin-α** and then incubated with 12 µM RCX for 48 h. The negative control (CTL) was treated with the vehicle (0.2% DMSO) used for diluting the tested compound. Doxorubicin (DOX, 2 µM) and oxaliplatin (OXA, 10 µM) **were used as positive controls.** Data are presented as the means ± S.E.M. of three independent experiments performed in duplicate. **For flow cytometry analysis,** 10,000 events were evaluated per experiment and cellular debris was omitted from the analysis. * *P* < 0.05 compared with the negative control by ANOVA, followed by the Student Newman-Keuls Test. # *P* < 0.05 compared with the respective treatment without inhibitor by ANOVA, followed by the Student Newman-Keuls Test.

**Table S1.** Selected ^1^H-NMR spectral data, δ ppm (J/Hz) of xanthoxylin, 1,10*'*-phenanthroline and *cis*-[Ru(phen)_2_(xant)](PF_6_) (RCX) in DMSO-*d*_6_.

| **H** | **Xanthoxylin** | **1,10***'***-phenanthroline** | **RCX** |
| --- | --- | --- | --- |
| 1-OH | δ 13.77 (*s*, 1H) | - | – |
| 6 | δ 6.11 (*dd*, J = 1.42, 2.20 Hz, 1H) | - | δ 5.66 (*d*, J = 2.03 Hz, 1H) |
| 4 | δ 6.07 (dd, 1.50, 2.38 Hz, 1H) | - | δ 5.59 (d, J = 2.31 Hz, 1H) |
| 5-OCH_3_ | δ 3.86 (d, J = 0.71 Hz, 3H) | - | δ 3.71 (s, 3H) |
| 3-OCH_3_ | δ 3.81 (d, J = 0.83 Hz, 3H) | - | δ 3.56 (s, 3H) |
| 2-COCH_3_ | δ 2.54 (d, J = 0.91, 3H) | - | δ 2.31 (s, 3H) |
| *a, a’* | - | δ 9.18 (dd, J = 2.51, 4.27 Hz, 2H) | δ 9.16 (t, J = 4.39 Hz, 1H) |
| *c, h* | - | δ 8.54 (dd, J = 6.30, 8.11 Hz, 2H) | δ 8.81 (t, J = 7.95 Hz, 2H) |
| *e,f, f’* | - | δ 8.02 (s, 1H) | δ 8.39 (m, 3H) |
| *i’* | - | δ 7.83 (dd, J = 3.79, 8.11 Hz, 2H) | δ 8.39 (m, 1H) |
| *h* | - | δ 8.02 (s, 1H) | δ 8.26 (m, 1H) |
| *i* | - | δ 7.83 (dd, J = 3.79, 8.11 Hz, 2H) | δ 8.26 (m, 1H) |
| *e’* | - | δ 8.02 (s, 1H) | δ 8.20 (m, 1H) |
| *j* | - | δ 9.18 (dd, J = 2.51, 4.27 Hz, 1H) | δ 8.20 (m, 1H) |
| *j’* | - | δ 9.18 (dd, J = 2.51, 4.27 Hz, 1H) | δ 7.97 (d, J = 5.12 Hz, 1H) |
| *c’* | - | δ 8.54 (dd, J = 6.30, 8.11 Hz, 1H) | δ 7.87 (d, J = 5.29 Hz, 1H) |
| *b, b’* | - | δ 7.83 (dd, J = 3.79, 8.11 Hz, 2H) | δ 7.46 (m, 2H) |

**Table S2.** The effect of the ruthenium complex with xanthoxylin (RCX) in gene expression of HepG2 cells

| Symbol | Full name | RQ | |
| --- | --- | --- | --- |
|  |  | DOX | RCX |
| ABL1 | ABL proto-oncogene 1, non-receptor tyrosine kinase | 2.628 | 1.908 |
| AKT1 | AKT serine/threonine kinase 1 | 1.813 | 1.685 |
| AKT2 | AKT serine/threonine kinase 2 | 3.275 | 2.697 |
| APC | APC, WNT signaling pathway regulator | 1.780 | 1.106 |
| BAX | BCL2 associated X, apoptosis regulator | 0.964 | 0.887 |
| BCAR1 | BCAR1, Cas family scaffolding protein | 2.176 | 2.846 |
| BCL2 | BCL2, apoptosis regulator | 1.140 | 2.377 |
| BCL2L1 | BCL2 like 1 | 0.663 | 0.898 |
| BCL2L11 | BCL2 like 11 | 13.988 | 9.786 |
| BID | BH3 interacting domain death agonist | 1.433 | 1.477 |
| BRAF | B-Raf proto-oncogene, serine/threonine kinase | 1.012 | 2.296 |
| CASP8 | caspase 8 | 1.414 | 1.493 |
| CASP9 | caspase 9 | 0.957 | N.d. |
| CCND1 | cyclin D1 | 0.820 | 4.752 |
| CCND2 | cyclin D2 | N.d. | N.d. |
| CCND3 | cyclin D3 | 1.151 | 2.397 |
| CCNE1 | cyclin E1 | 1.535 | 0.674 |
| CDC42 | cell division cycle 42 | 1.077 | 4.147 |
| CDH1 | cadherin 1 | N.d. | 1.051 |
| CDK2 | cyclin dependent kinase 2 | 4.331 | 1.251 |
| CDK4 | cyclin dependent kinase 4 | 1.427 | 2.737 |
| CDKN1A | cyclin dependent kinase inhibitor 1A | 2.849 | 0.628 |
| CDKN1B | cyclin dependent kinase inhibitor 1B | 1.029 | 0.543 |
| CDKN2A | cyclin dependent kinase inhibitor 2A | 2.027 | 3.836 |
| CDKN2B | cyclin dependent kinase inhibitor 2B | N.d. | N.d. |
| COL1A1 | collagen type I alpha 1 chain | N.d. | N.d. |
| CRK | CRK proto-oncogene, adaptor protein | 2.519 | 6.172 |
| CTNNB1 | catenin beta 1 | 2.392 | 1.930 |
| CYCS | cytochrome c, somatic | 0.582 | 1.927 |
| DVL1 | dishevelled segment polarity protein 1 | 0.539 | 2.712 |
| E2F1 | E2F transcription factor 1 | 4.768 | 7.081 |
| EGFR | epidermal growth factor receptor | 0.502 | 1.985 |
| ELK1 | ELK1, ETS transcription factor | 2.142 | 5.179 |
| ERBB2 | erb-b2 receptor tyrosine kinase 2 | 1.746 | 2.186 |
| FADD | Fas associated via death domain | 3.100 | 1.854 |
| FAS | Fas cell surface death receptor | 2.459 | 2.845 |
| FASLG | Fas ligand | N.d. | N.d. |
| FGF2 | fibroblast growth factor 2 | 1.914 | 5.503 |
| FN1 | fibronectin 1 | N.d. | N.d. |
| FOS | Fos proto-oncogene, AP-1 transcription factor subunit | 1.514 | 1.472 |
| FYN | FYN proto-oncogene, Src family tyrosine kinase | 1.034 | 2.240 |
| FZD1 | frizzled class receptor 1 | 5.811 | 4.807 |
| GRB2 | growth factor receptor bound protein 2 | 1.030 | 0.855 |
| GSK3B | glycogen synthase kinase 3 beta | 0.590 | 0.999 |
| HGF | hepatocyte growth factor | N.d. | N.d. |
| HRAS | HRas proto-oncogene, GTPase | 1.370 | 2.071 |
| IGF1 | insulin like growth factor 1 | N.d. | N.d. |
| IGF1R | insulin like growth factor 1 receptor | 1.160 | 1.052 |
| ITGA2B | integrin subunit alpha 2b | 2.384 | 5.437 |
| ITGAV | integrin subunit alpha V | 2.101 | 1.126 |
| ITGB1 | integrin subunit beta 1 | 1.941 | 1.547 |
| ITGB3 | integrin subunit beta 3 | 1.707 | 1.478 |
| JUN | Jun proto-oncogene, AP-1 transcription factor subunit | 7.340 | 4.475 |
| KDR | kinase insert domain receptor | N.d. | N.d. |
| KIT | KIT proto-oncogene receptor tyrosine kinase | N.d. | N.d. |
| KRAS | KRAS proto-oncogene, GTPase | 2.552 | 4.786 |
| LEF1 | lymphoid enhancer binding factor 1 | N.d. | N.d. |
| MAP2K1 | mitogen-activated protein kinase kinase 1 | 2.441 | 2.591 |
| MAP3K5 | mitogen-activated protein kinase kinase kinase 5 | 1.887 | 1.752 |
| MAPK1 | mitogen-activated protein kinase 1 | 0.857 | 2.124 |
| MAPK14 | mitogen-activated protein kinase 14 | 2.163 | 3.025 |
| MAPK3 | mitogen-activated protein kinase 3 | 1.561 | 1.762 |
| MAPK8 | mitogen-activated protein kinase 8 | 1.273 | 1.293 |
| MAX | MYC associated factor X | N.d. | N.d. |
| MDM2 | MDM2 proto-oncogene | 0.928 | 3.153 |
| MYC | MYC proto-oncogene, bHLH transcription factor | 0.392 | 4.304 |
| NFKB1 | nuclear factor kappa B subunit 1 | 2.017 | 2.156 |
| NFKB2 | nuclear factor kappa B subunit 2 | 3.594 | 3.621 |
| NFKBIA | NFKB inhibitor alpha | 1.224 | 1.342 |
| NRAS | NRAS proto-oncogene, GTPase | 1.874 | 1.862 |
| PIK3CA | phosphatidylinositol-4,5-bisphosphate 3-kinase catalytic subunit alpha | 2.854 | 1.265 |
| PIK3R1 | phosphoinositide-3-kinase regulatory subunit 1 | 6.207 | 1.408 |
| PTEN | phosphatase and tensin homolog | 0.476 | 0.644 |
| PTK2 | protein tyrosine kinase 2 | 0.948 | 2.034 |
| PTK2B | protein tyrosine kinase 2 beta | 0.530 | 0.803 |
| RAC1 | ras-related C3 botulinum toxin substrate 1 (rho family, small GTP binding protein Rac1) | 2.048 | 1.199 |
| RAF1 | Raf-1 proto-oncogene, serine/threonine kinase | 3.447 | 4.645 |
| RB1 | RB transcriptional corepressor 1 | 1.230 | 0.795 |
| RELA | RELA proto-oncogene, NF-kB subunit | 1.074 | 2.403 |
| RHOA | ras homolog family member A | 1.270 | 1.541 |
| SHC1 | SHC adaptor protein 1 | 1.865 | 3.702 |
| SMAD4 | SMAD family member 4 | 2.601 | 3.549 |
| SOS1 | SOS Ras/Rac guanine nucleotide exchange factor 1 | 2.199 | 4.992 |
| SPP1 | secreted phosphoprotein 1 | N.d. | N.d. |
| SRC | SRC proto-oncogene, non-receptor tyrosine kinase | 1.036 | 1.833 |
| TCF3 | transcription factor 3 | 1.915 | 3.166 |
| TGFB1 | transforming growth factor beta 1 | 1.413 | 1.267 |
| TGFBR1 | transforming growth factor beta receptor 1 | 1.403 | 1.352 |
| TGFBR2 | transforming growth factor beta receptor 2 | 1.094 | 1.069 |
| TP53 | tumor protein p53 | 0.945 | 0.471 |
| VEGFA | vascular endothelial growth factor A | 1.772 | 3.759 |
| WNT1 | Wnt family member 1 | N.d. | N.d. |

HepG2 cells were treated with 12 µM RCX for 12 h. The negative control was treated with the vehicle (0.2% DMSO) used for diluting the tested compound. Doxorubicin (DOX, 2 µM) was used as a positive control. After treatment, total RNA was isolated and reverse transcribed. Gene expression was detected using the 96-well plate TaqMan® Array Human Molecular Mechanisms of Cancer. GAPDH, 18S and HPRT1 genes were used as endogenous genes for normalization. Values represent the relative quantitation (RQ) compared with the calibrator (cells treated with the negative control, RQ = 1.0).

**Table S3.** Effect of the ruthenium complex with xanthoxylin (RCX) on body and relative organ weight from C.B-17 SCID mice bearing HepG2 cells

| **Parameters** | **CTL** | **DOX** | **5-FU** | **RCX** | **RCX** |
| --- | --- | --- | --- | --- | --- |
| Dose (mg/kg) | - | 0.3 | 10 | 2.5 | 5 |
| Survival | 15/15 | 14/14 | 9/15 | 14/14 | 7/14 |
| Initial body weight (g) | 21.2 ± 0.4 | 22.3 ± 0.4 | 25.2 ± 0.4 | 22.2 ± 0.5 | 21.1 ± 0.4 |
| Final body weight (g) | 20.4 ± 0.3 | 19.5 ± 0.6 | 24.1 ± 1.7 | 20.6 ± 0.4 | 17.0 ± 0.4* |
| Liver (g/100 g body weight) | 5.0 ± 0.1 | 4.8 ± 0.1 | 3.4 ± 0.7 | 4.9 ± 0.2 | 4.2 ± 0.4 |
| Kidney (g/100 g body weight) | 1.5 ± 0.04 | 1.5 ± 0.04 | 1.2 ± 0.4 | 1.6 ± 0.04 | 1.5 ± 0.06 |
| Heart (g/100 g body weight) | 0.5 ± 0.01 | 0.5 ± 0.02 | 0.5 ± 0.2 | 0.5 ± 0.02 | 0.6 ± 0.03 |
| Lung (g/100 g body weight) | 0.9 ± 0.07 | 0.9 ± 0.06 | 0.6 ± 0.2 | 0.8 ± 0.04 | 0.8 ± 0.04 |

The negative control (CTL) was treated with the vehicle (5% DMSO) used for diluting the tested compound. Doxorubicin (DOX) and 5-fluorouracil (5-FU) were used as positive controls. Beginning 1 day after tumor implantation, the animals were treated through the intraperitoneal route for 21 consecutive days. Data are presented as the means ± S.E.M. of 7-15 animals. * *P* < 0.05 compared with the negative control by ANOVA, followed by the Student Newman-Keuls Test.

.

**Table S4.** Effect of the ruthenium complex with xanthoxylin (RCX) on hematological parameters of peripheral blood from C.B-17 SCID mice bearing HepG2 cells

| **Parameters** | **Non-tumor** | **CTL** | **DOX** | **5-FU** | **RCX** | **RCX** |
| --- | --- | --- | --- | --- | --- | --- |
| Dose (mg/kg) | - | - | 0.3 | 10 | 2.5 | 5 |
| Erythrocytes  (10^6^cells/μL) | 11.4 ± 0.3 | 8.1 ± 0.7 | 8.9 ± 0.7 | 8.4 ± 0.5 | 9.0 ± 0.6 | 8.3 ± 0.3 |
| Total leukocytes (10^3^cells/μL) | 3.1 ± 0.5 | 3.6 ± 0.8 | 1.5 ± 0.2* | 1.8 ± 0.3* | 3.1 ± 0.5 | 2.6 ± 0.2 |
| Differential leukocytes (%) | |  |  |  |  |  |
| Neutrophils | 41.0 | 59.7 | 48.8 | 65.0 | 48.3 | 49.0 |
| Lymphocytes | 53.8 | 22.0 | 49.3 | 32.8 | 49.3 | 49.0 |
| Monocytes | 3.2 | 0.8 | 1.0 | 1.3 | 1.0 | 1.0 |
| Eosinophils | 1.2 | 0.8 | 1.0 | 1.0 | 1.0 | 1.0 |

Non-tumor group represents C.B-17 SCID mice without tumor inoculation or any treatment. The negative control (CTL) was treated with the vehicle (5% DMSO) used for diluting the tested compound. Doxorubicin (DOX) and 5-fluorouracil (5-FU) was used as positive controls. Beginning 1 day after tumor implantation, the animals were treated through the intraperitoneal route for 21 consecutive days. Data are presented as the means ± S.E.M. of 5-6 animals. * *P* < 0.05 compared with the negative control by ANOVA, followed by the Student Newman-Keuls Test.

**Table S5.** List of cell lines used, their histological type, origin and source of cells.

| **Cell line** | **Histological type** | **Origin** | **Source** |
| --- | --- | --- | --- |
| Cancer cells |  |  |  |
| HCT116 | colon carcinoma | human | ATCC |
| HT-29 | colon adenocarcinoma | human | ATCC |
| MCF-7 | breast adenocarcinoma | human | ATCC |
| HepG2 | hepatocellular carcinoma | human | ATCC |
| HSC-3 | oral squamous cell carcinoma | human | ATCC |
| SCC-4 | oral squamous cell carcinoma | human | ATCC |
| SCC-9 | oral squamous cell carcinoma | human | ATCC |
| SCC-15 | oral squamous cell carcinoma | human | ATCC |
| SCC-25 | oral squamous cell carcinoma | human | ATCC |
| AGP-01 | ascitic gastric adenocarcinoma | human | UFPA |
| ACP-02 | gastric adenocarcinoma | human | UFPA |
| ACP-03 | gastric adenocarcinoma | human | UFPA |
| HL-60 | promyelocytic leukemia | human | ATCC |
| K-562 | chronic myelogenous leukemia | human | ATCC |
| B16-F10 | melanoma | mouse | ATCC |
|  |  |  |  |
| Non-cancer cells |  |  |  |
| MRC-5 | lung fibroblast | human | ATCC |
| HaCAT | keratinocyte | human | ATCC |
| PBMC | peripheral blood mononuclear cells | human | primary cell culture |
|  |  |  |  |
| Mutant and its parental cell | |  |  |
| BAD KO SV40 MEF | immortalized mouse embryonic fibroblasts with the BAD gene knocked out | mouse | ATCC |
| WT SV40 MEF | wild-type immortalized embryonic fibroblasts | mouse | ATCC |

ATCC denotes American Type Culture Collection and UFPA denotes Federal University of Para, PA, Brazil (kindly donated by Dr. Raquel Carvalho Montenegro and Dr. Rommel Mario Rodriguez Burbano). Primary cell culture of Peripheral blood mononuclear cells (PBMCs) were obtained by standard ficoll density protocol. The Research Ethics Committee of the Oswaldo Cruz Foundation (Salvador, Bahia, Brazil) approved the experimental protocol (# 031019/2013).
